# Enhancing protein expression in humans through codon optimization with transformer and contrastive learning

**DOI:** 10.1016/j.omtn.2026.102991

**Published:** 2026-06-18

**Authors:** Juseong Kim, Jeongmu Kim, Jae-Wook Lee, Qian Qi, Caroline Danehy, Ho Young Kang, Yong Cheng, Giltae Song

**Affiliations:** 1 Division of Artificial Intelligence, Pusan National University, Busan 46241, South Korea; 2 Center for Artificial Intelligence Research, Pusan National University, Busan 46241, South Korea; 3 School of Computer Science and Engineering, Pusan National University, Busan 46241, South Korea; 4 Research & Development, NuclixBio, Seoul 08377, South Korea; 5 Department of Hematology MS 341, St. Jude Children’s Research Hospital, 262 Danny Thomas Place, Memphis, TN 38105, USA

**Keywords:** MT: Bioinformatics, codon optimization, deep learning, contrastive learning, protein expression, stability, *Homo sapiens*

## Abstract

Following the success of COVID-19 vaccines and therapies, mRNA-based therapeutics have attracted significant attention. Codon optimization is crucial for improving translation efficiency and mRNA stability, both of which are necessary for developing effective mRNA vaccines and drugs. Traditional approaches often rely on codon usage tables and other frequency-based heuristics, which neglect sequence context and show limited scalability. We introduce COformer, a deep learning framework for codon optimization that captures codon-level features related to protein expression in *Homo sapiens* cells. By integrating convolutional neural networks (CNNs) and transformers, the model learns local and global sequence contexts influencing translation efficiency and stability, while contrastive learning aligns codon representations with amino acid identities. Trained on sequences optimized by commercial tools, COformer generates codon choices that, when validated in *in vitro* experiments, enhance protein expression through optimized codon usage and improved mRNA stability.

## Graphical abstract

**Figure:**
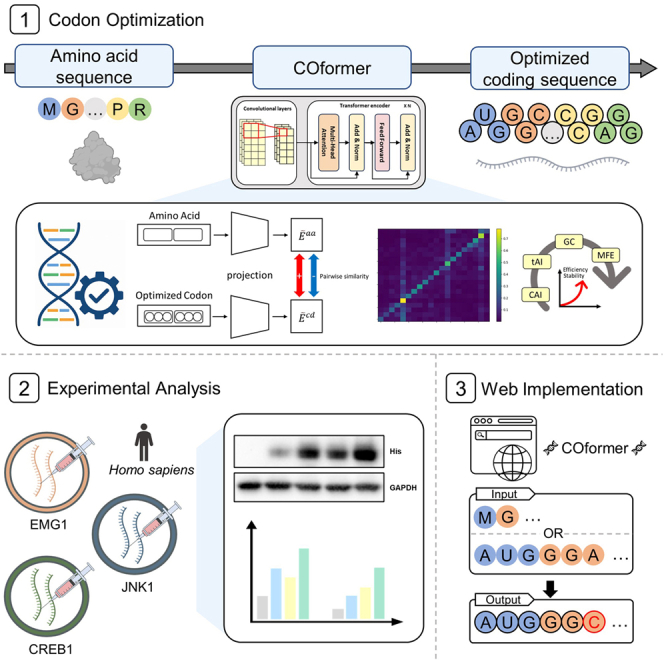

## Teaser

COformer is a deep learning framework for codon optimization in mammalian expression systems. By learning codon-selection patterns from diverse optimization strategies, it captures context-dependent sequence features beyond conventional codon metrics and enables expression-oriented synonymous coding sequence design across multiple target genes.

## Introduction

Achieving optimal and safe transgene expression remains a key challenge in gene and mRNA therapeutics. The efficacy of gene therapy often hinges on delivering sufficient amounts of therapeutic protein to correct or compensate for genetic defects. Conventionally, this has been addressed through the use of strong enhancers and promoters to drive robust expression. However, these strategies have inherent limitations. Potent enhancers pose risks of genotoxicity by aberrantly activating neighboring genes, especially in integrating viral vectors.^1^ Furthermore, in clinically relevant cell types such as hematopoietic stem and progenitor cells (HSPCs), many widely used promoters like CMV are prone to silencing, necessitating reliance on more stable but weaker promoters such as the elongation factor 1-alpha (EF1a) promoter.^2^^,^^3^^,^^4^^,^^5^ This constraint reduces flexibility in modulating protein output and highlights a critical need for orthogonal strategies that can fine-tune gene expression post-transcriptionally.

Codon optimization offers a promising solution to this challenge by enabling control at the coding-sequence level.^6^^,^^7^ Historically, codon optimization has focused on improving translational efficiency, reflecting the strong influence of codon usage bias and transfer RNA (tRNA) availability on ribosome loading and elongation dynamics.^6^ In many biological contexts, enhanced translational efficiency is beneficial because efficient ribosome loading increases the rate of protein synthesis from a given transcript. Beyond translational efficiency, mRNA structural stability represents an additional post-transcriptional determinant of protein output.^8^ Transcript lifetime directly constrains the number of translation cycles an mRNA can undergo, and increased stability has been shown to synergize with efficient translation to support repeated rounds of protein synthesis prior to decay.^9^ Accordingly, effective codon optimization often considers both how efficiently an mRNA is translated and how long it persists in the cellular environment.

Originally developed for bacterial expression systems, codon optimization has since become essential in modern biotechnology and mRNA therapeutics, where regulatory control operates downstream of transcription. In contrast to DNA-based expression systems, where protein yield can be modulated at the transcriptional level through promoter and enhancer elements, mRNA-based therapeutics rely primarily on post-transcriptional properties of the mRNA molecule itself, including features encoded within the coding sequence. This design paradigm has been exemplified by mRNA vaccine platforms, in which codon optimization has been widely used as part of sequence-engineering strategies to enhance antigen expression in mRNA vaccine platforms, including COVID-19 vaccines.^10^ Because codon modification acts downstream of transcription, it can augment protein expression without altering promoter architecture or enhancer elements.^11^^,^^12^

In addition to these regulatory considerations, mRNA-based therapeutics offer practical advantages at the production level, as mRNA molecules can be rapidly synthesized via *in vitro* transcription, enabling fast responses to emerging diseases and personalized therapies.^11^^,^^12^ Despite these advantages, mRNA therapeutics remain challenged by transcript instability and suboptimal protein expression, underscoring the need for robust sequence engineering approaches.^13^ Codon optimization algorithms address these challenges by enabling the systematic redesign of mRNA sequences to balance multiple objectives, including translational efficiency and mRNA stability,^14^^,^^15^^,^^16^ as well as reduced immunogenicity and efficient RNA synthesis in therapeutic contexts,^17^ providing a robust foundation for the development of safe and effective mRNA therapeutics.

Although synonymous codon substitutions are often discussed in the context of translational efficiency and mRNA stability, accumulating evidence indicates that they may also influence broader regulatory processes, including pre-mRNA splicing, miRNA binding, and the generation of aberrant or unwanted transcripts.^18^ Acknowledging these broader effects, the present study focuses on the role of synonymous codon choice in shaping translational efficiency and mRNA stability within codon optimization frameworks.

However, conventional optimization strategies typically rely on in-depth biological knowledge of the host’s translational machinery, including tRNA abundance,^19^ codon usage bias, and mRNA stability. Codon usage bias refers to genome- or host-specific preferences in synonymous codon usage and is often quantified using codon usage frequencies or indices such as the codon adaptation index (CAI), which measures the similarity of a gene’s codon usage to that of highly expressed genes in a given host.^20^ mRNA stability is commonly assessed using metrics such as minimum free energy (MFE),^21^ a thermodynamic proxy for predicted RNA secondary-structure stability, where lower values generally indicate more stable structures.^22^^,^^23^ These approaches also incorporate additional structural and sequence-level constraints.^7^

While effective in bacterial systems, these methods often fall short in mammalian contexts due to their static, heuristic nature, which cannot capture complex sequence context or higher-order codon dependencies,^24^^,^^25^ reducing their adaptability in evolving therapeutic settings.

Recent advances in computational biology, particularly through the integration of machine learning techniques, have led to the development of novel methods that significantly enhance traditional practices.^26^^,^^27^ Deep learning, known for its ability to model complex relationships within biological data, has shown promise in optimizing codon usage.^28^^,^^29^^,^^30^^,^^31^ However, existing models are often trained with a primary focus on specific optimization objectives, such as codon usage bias or sequence distributions, which can limit their robustness when applied across diverse genetic contexts. Moreover, systematically integrating additional regulatory features—such as GC content, ribosome binding sites, and mRNA structural elements—into a unified optimization framework remains challenging.^32^^,^^33^^,^^34^ These limitations highlight the need for more flexible and data-driven approaches that can dynamically account for the intricacies of translational regulation.

To address these limitations, we introduce COformer, a codon optimization framework designed for *Homo sapiens*. COformer integrates convolutional neural networks (CNNs) and transformer encoders to model sequence context at multiple scales. Here, we use local context to denote short-range codon-context patterns (e.g., codon-pair and motif-level dependencies) that have been reported to associate with translation-related outcomes in specific biological or experimental contexts.^8^^,^^35^

We use global context to denote longer-range sequence features spanning the coding region, such as compositional patterns (e.g., GC distribution and codon-position-dependent periodicity across GC1, GC2, and GC3) and predicted RNA-structural tendencies. Importantly, these terms describe patterns in the nucleotide or codon sequence and commonly used proxy features, rather than direct mechanistic determinants of translational regulation, such as tRNA-modification-dependent decoding. Consistent with our goal of improving protein output in human cells, COformer leverages these multi-scale sequence representations to generate synonymous designs that perform well in the experimental setting evaluated in this study.

Furthermore, the model incorporates contrastive learning to encourage representation consistency between amino acid inputs and codon outputs, ensuring that synonymous codons encoding the same amino acid are embedded in a way that reflects their functional similarity, enabling COformer to move beyond static frequency tables and learn codon patterns associated with high protein yield.^36^^,^^37^

Distinct from conventional models, COformer is trained on sequences pre-optimized by multiple commercial tools, not as biologically validated optima, but as heuristic approximations reflecting practical design constraints commonly used in codon optimization.^38^^,^^39^^,^^40^ In the absence of a definitive ground truth for synonymous codon selection, this strategy exposes the model to a diverse empirical design space rather than a single, assumption-driven optimization rule.^6^^,^^34^ By learning recurrent codon selection patterns across heterogeneous-tool-generated sequences, COformer aims to capture context-dependent regularities in codon usage without presuming universal biological optimality. This data-driven formulation enables flexible representation learning while remaining agnostic to any single underlying optimization hypothesis.

We demonstrate that COformer consistently improves several commonly used sequence-level metrics, including CAI, MFE, and GC content.^20^^,^^22^^,^^41^ These metrics provide useful proxies for codon usage and predicted RNA structural properties, but they should not be interpreted as direct predictors of protein expression. More importantly, we experimentally evaluate COformer-designed sequences in HEK293T cells and observe up to an 11.3-fold increase in protein expression over their corresponding wild-type sequences under the tested conditions.^42^ These findings position COformer as a data-driven and experimentally benchmarked framework for optimizing transgene expression in therapeutic gene design.

By addressing current limitations in regulatory element usage and enabling expression tuning at the translational level, COformer complements existing gene therapy platforms and provides a scalable, flexible tool for synthetic gene design in human cells.^5^^,^^43^

## Results

### Learning from diverse synonymous codon landscapes

**Figure 1:**
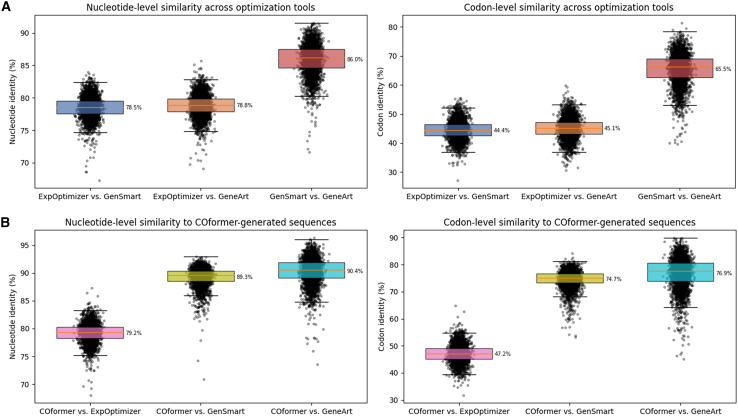
Pairwise sequence similarity among codon-optimized coding sequences (A) Pairwise nucleotide-level and codon-level identity among coding sequences generated by ExpOptimizer, GenSmart, and GeneArt for identical protein inputs. Nucleotide-level identity was calculated from aligned coding sequences, and codon-level identity was calculated as the fraction of codon positions with identical codon choices. (B) Nucleotide-level and codon-level identity between COformer-generated sequences and each commercial tool output on the same held-out test proteins. Each point represents one protein comparison, and boxplots summarize the distribution across the test set. The center line indicates the median, the box spans the interquartile range, and whiskers extend to the most extreme values within 1.5 times the interquartile range.

To relate COformer designs to existing practice, we first quantified how closely widely used commercial optimizers agree on synonymous solutions for the same proteins and then assessed how COformer outputs align with each platform. Because COformer was trained using tool-optimized coding sequences, we focused on three widely used commercial codon optimization platforms—NovoPro ExpOptimizer, GenScript GenSmart, and Thermo Fisher GeneArt—as reference baselines for comparison^38^^,^^39^^,^^40^ and asked whether these tools converge on similar synonymous designs for identical proteins. Across tool pairs, nucleotide-level identity was moderately high, with median values ranging from 78.5% to 86.0%, consistent with constraints imposed by the fixed amino acid sequence. In contrast, codon-level identity was substantially lower, with median values ranging from 44.4% to 65.5% across tool pairs (Figure 1A), indicating that the platforms often select different synonymous codons despite encoding the same proteins. GenSmart and GeneArt were more similar to each other than either was to ExpOptimizer, suggesting more closely aligned heuristic preferences.

We next compared COformer-generated sequences with outputs from each commercial tool on the same held-out proteins. COformer designs were most similar to GeneArt at the nucleotide level, with a median identity of 90.4%, and showed higher codon-level similarity to GeneArt and GenSmart than to ExpOptimizer, with median identities of 76.9%, 74.7%, and 47.2%, respectively (Figure 1B). These similarity patterns suggest that the training targets capture distinct, tool-dependent codon-selection regimes. At the same time, COformer generates synonymous designs that remain within this heterogeneous design space rather than reproducing the codon choices of a single commercial platform.

### Experimental validation of COformer designs in human cells

To evaluate whether COformer designs improve protein output in human cells, we selected three human genes (*EMG1*, *JNK1*, and *CREB1*) spanning distinct functional classes and regulatory contexts. EMG1 is required for ribosome biogenesis and 18S rRNA methylation and is linked to Bowen-Conradi syndrome.^44^ c-Jun N-terminal kinase 1 (JNK1) participates in the mitogen-activated protein kinase (MAPK) stress-response pathway and regulates apoptosis and inflammation.^45^ CREB1 is a transcription factor implicated in neuronal development, memory consolidation, and circadian rhythm regulation.^46^ The selected genes exhibited relatively low baseline codon adaptation characteristics, making them suitable for evaluating whether COformer could improve protein expression relative to both wild-type sequences and existing commercial optimization methods across distinct sequence contexts. To evaluate generalization to unseen targets, none of the genes used for experimental validation (*EMG1*, *JNK1*, and *CREB1*) were included in the training dataset.

**Figure 2:**
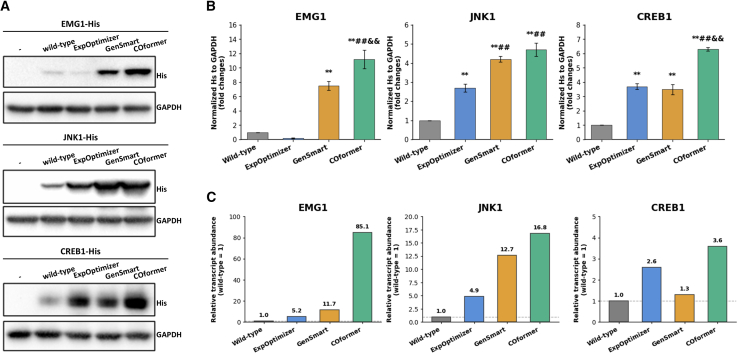
Experimental comparison of codon-optimized constructs in HEK293T cells (A) Western blot analysis of HEK293T cells transfected with wild-type or codon-optimized *EMG1*, *JNK1*, and *CREB1* constructs generated by ExpOptimizer, GenSmart, or COformer. Protein expression was detected using an anti-His antibody, with GAPDH as a loading control. (B) Quantification of protein expression normalized to GAPDH and shown as fold change relative to wild-type. Data represent mean ± SD from three independent experiments. Statistical significance was assessed using one-way ANOVA followed by Tukey’s multiple comparison test. ∗*p* < 0.05, ∗∗*p* < 0.01 vs. wild-type; #*p* < 0.05, ##*p* < 0.01 vs. ExpOptimizer; &*p* < 0.05, &&*p* < 0.01 vs. GenSmart. (C) Relative transcript abundance measured by RNA-seq 24 h post-transfection and normalized to *GAPDH*. Expression values are shown as fold change relative to wild type.

We performed transient transfection experiments in HEK293T cells to enable controlled comparison across synonymous variants under consistent delivery conditions. For each gene, we designed four coding-sequence variants: the wild-type sequence and sequences optimized by ExpOptimizer, GenSmart, and COformer. The corresponding coding regions were synthesized, cloned into a mammalian expression vector (pAcGFP1-N1), and expressed as C-terminal 6×His-tagged proteins. Protein levels were assessed by western blot using an anti-His antibody, with GAPDH as a loading control, 24 h post-transfection (Figure 2A). Original western blot images are provided in Figure S1.

To further examine whether differences in protein output were associated with changes in transcript abundance, total RNA was extracted from transfected cells 24 h post-transfection and analyzed by RNA sequencing (RNA-seq). Transcript abundance for each synonymous construct was quantified using kallisto with a custom reference containing all construct sequences together with *GAPDH* as an internal normalization control.^47^ Because synonymous variants of the same gene share extensive sequence similarity, background correction was applied to reduce cross-mapping artifacts among related constructs.

COformer designs yielded higher protein expression than the other variants across all three genes (Figure 2B). COformer increased EMG1 expression by 11.3-fold relative to wild type and also exceeded ExpOptimizer- and GenSmart-optimized designs (28.1- and 1.5-fold, respectively). JNK1 and CREB1 showed similar patterns, with increases of 4.8- and 6.3-fold relative to wild type, respectively, along with additional gains over ExpOptimizer (1.8- and 1.7-fold, respectively) and GenSmart (1.1- and 1.8-fold, respectively). Statistical analysis using one-way ANOVA followed by Tukey’s multiple comparison test confirmed significant differences among codon optimization methods across the tested genes.

RNA-seq analysis revealed that COformer constructs also exhibited increased transcript abundance relative to wild type across all three genes (Figure 2C). In particular, EMG1 showed a marked increase in transcript levels for the COformer construct compared with the other synonymous variants, consistent with the strong enhancement observed at the protein level. These results suggest that improved protein expression by COformer may be associated, at least in part, with increased steady-state transcript abundance in human cells.

Notably, the ExpOptimizer-optimized EMG1 construct exhibited lower protein expression than wild type, unlike the trends observed for JNK1 and CREB1. This result suggests that the effects of codon optimization can be gene dependent and that a given optimization strategy does not necessarily improve protein output for every target sequence.

Together, these experiments provide experimental support that COformer-generated synonymous designs can enhance both transcript abundance and protein output in HEK293T cells under the conditions tested here.

### Codon-usage and codon-bias metrics across optimization methods

**Figure 3:**
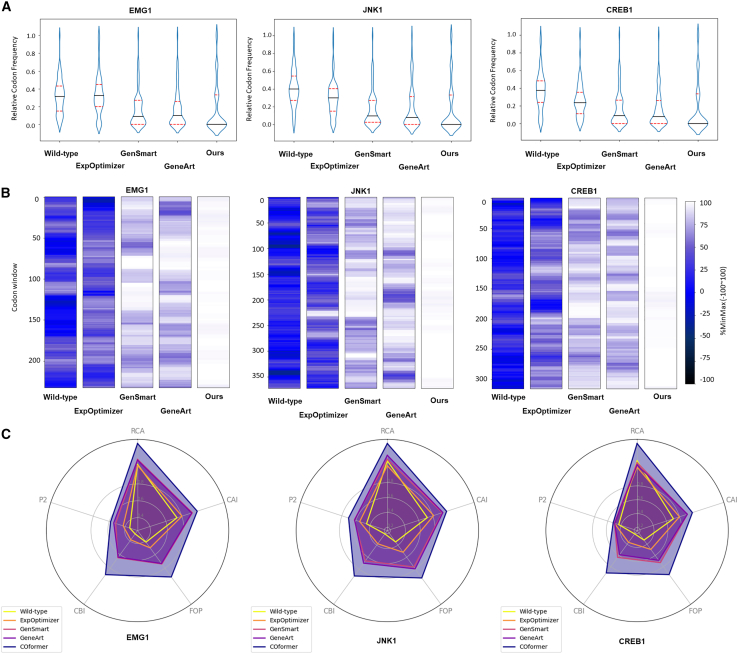
Codon-usage and codon-bias metrics across optimization methods (A) Distribution of relative codon frequencies for wild-type, ExpOptimizer, GenSmart, and COformer sequences across *EMG1*, *JNK1*, and *CREB1*. Relative codon frequency was calculated within each synonymous codon family as the fraction assigned to each codon for a given amino acid. Violin plots show the distribution of relative codon frequencies, with internal lines indicating the 25th percentile, median, and 75th percentile. (B) Sliding-window %MinMax profiles showing local codon preference along each coding sequence. %MinMax was calculated relative to the human reference codon-usage table using a sliding codon window. (C) Summary of codon-usage and codon-bias metrics, including RCA, CAI, FOP, CBI, and P2.

To further examine codon-usage patterns across the selected genes, we analyzed the distribution of relative codon frequencies (Figure 3A). Compared with wild-type and commercial tool outputs, COformer designs showed reduced dispersion in relative codon-frequency profiles across *EMG1*, *JNK1*, and *CREB1*, suggesting less variable codon-frequency patterns across synonymous positions.^48^^,^^49^

To assess local codon-usage regularity along coding sequences, we analyzed %MinMax profiles using sliding codon windows (Figure 3B). %MinMax quantifies local codon choice relative to preferred and non-preferred synonymous alternatives in a host reference set.^50^ COformer-generated sequences exhibited smoother %MinMax profiles along coding regions than wild-type and commercial tool outputs.

We also evaluated codon usage and bias using five complementary metrics—RCA,^51^ CAI,^20^ FOP,^52^ CBI,^53^ and P2 (Figure 3C).^54^ While these metrics are partially correlated and broadly reflect codon usage preferences, they capture distinct aspects of codon bias, including adaptation to host-preferred codons (CAI and RCA), enrichment of optimal codons (FOP and CBI), and directional codon bias (P2). Across these complementary measures, COformer designs consistently exhibited higher values than both the wild-type and commercial tool outputs, indicating a shift toward codon-usage patterns that are more consistent with the selected host reference. P2 was originally introduced in bacterial codon-usage analyses as an index related to biased third-position pyrimidine usage and translational selection.^54^^,^^55^ Because this index was not developed specifically for mammalian therapeutic sequence design, we used P2 only as a descriptive codon-bias indicator rather than as a direct predictor of protein expression.

#### Nucleotide composition and predicted RNA secondary-structure features

**Figure 4:**
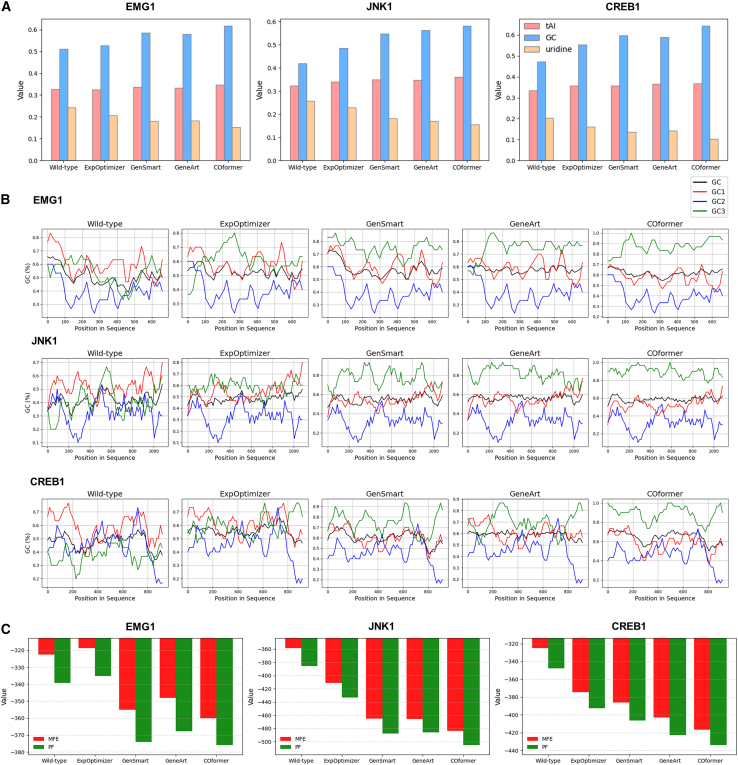
Nucleotide composition and predicted RNA secondary-structure features (A) tRNA adaptation index (tAI), GC fraction, and uridine fraction for wild-type, ExpOptimizer, GenSmart, and COformer sequences. (B) Sliding-window GC content by codon position, including GC1, GC2, and GC3. (C) Minimum free energy (MFE) and partition-function (PF) values computed using ViennaRNA.

We next analyzed composition- and structure-related metrics for *EMG1*, *JNK1*, and *CREB1* (Figure 4). Figure 4A summarizes tRNA adaptation index (tAI), GC fraction, and uridine fraction. Across all three genes, COformer designs showed higher tAI values than wild-type and commercial tool outputs, suggesting higher predicted tRNA adaptation. While tAI is often used as a proxy for decoding efficiency, this relationship can be context-dependent in mammalian systems.^56^ COformer designs also showed moderated uridine fractions, which have been associated with stability- and sensing-related properties in some settings.^19^^,^^57^^,^^58^^,^^59^

Figure 4B shows sliding-window GC content by codon position (GC1, GC2, and GC3). As expected, GC1 and GC2 are strongly constrained by amino acid identity, whereas GC3 varies across methods due to synonymous codon choice. In particular, GC2 is closely associated with amino acid identity and chemical properties and therefore primarily reflects protein-level constraints rather than codon-level optimization. Accordingly, variation in GC1 and GC2 provides limited additional insight into synonymous codon usage. COformer-generated sequences showed more consistent GC3 profiles relative to wild-type and commercial tools, consistent with controlled synonymous codon selection while preserving amino acid sequence constraints.^60^^,^^61^

To assess predicted RNA structural properties, we computed minimum free energy (MFE) and partition-function (PF) values for coding sequences using ViennaRNA (Figure 4C).^22^^,^^62^ COformer designs showed lower MFE values across the examined genes, reflecting greater predicted RNA secondary-structure stability at the sequence level. PF values showed a similar directional trend. Together, these metrics suggest that COformer alters codon usage and nucleotide composition in ways that influence predicted RNA folding tendencies, consistent with sequence features commonly considered in codon optimization.

Because increased secondary-structure stability near the 5′ region can impede translation initiation,^8^ we additionally examined local folding trends using sliding-window MFE profiles calculated from the start codon of the coding sequence. Across multiple window sizes (30, 60, 90, and 120 nt), COformer-generated sequences did not show strong 5′-localized decreases in MFE relative to other methods (Figures S2–S4). This may partly reflect the GC-rich composition commonly observed near the 5′ regions of human protein-coding genes and the contribution of GC-rich codon positions to predicted coding-region RNA secondary structure.^63^^,^^64^^,^^65^ These results suggest that the lower global MFE values produced by COformer were not solely driven by pronounced 5′-localized stabilization.

### Benchmarking COformer against existing codon optimization models and tools

We next benchmarked COformer against three commercial codon-optimization tools (ExpOptimizer, GenSmart, and GeneArt) and two learning-based baselines (ICOR and CodonTransformer) on the same held-out test set.^29^^,^^31^^,^^38^^,^^39^^,^^40^ Because codon optimization does not yield a single ground-truth target sequence, we evaluated methods using commonly reported sequence-level descriptors of codon usage, nucleotide composition, and predicted structure.

**Figure 5:**
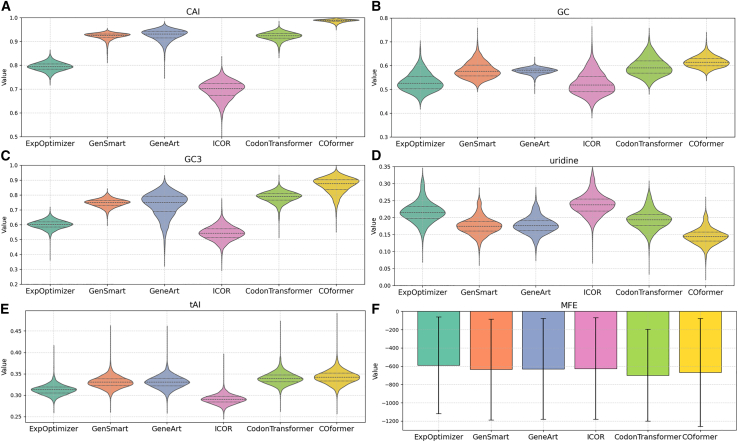
Benchmarking COformer against commercial tools and learning-based models on a held-out test set COformer was compared with ExpOptimizer, GenSmart, GeneArt, ICOR, and CodonTransformer using identical held-out protein inputs. Sequence-level descriptors included (A) CAI, (B) overall GC fraction, (C) GC3 fraction, (D) uridine fraction, and (E) tAI. For (A–E), each distribution represents sequence-level values calculated for individual held-out protein inputs. Violin width reflects the density of observations, and internal lines indicate the 25th percentile, median, and 75th percentile. (F) Predicted RNA secondary-structure MFE was computed using ViennaRNA. For the boxplot, the center line indicates the median, the box spans the interquartile range, and whiskers extend to the most extreme values within 1.5 times the interquartile range.

Specifically, we computed codon adaptation index (CAI), tRNA adaptation index (tAI), overall GC fraction, GC content at the third codon position (GC3), uridine fraction, and minimum free energy (MFE) of predicted RNA secondary structure (Figure 5). These metrics provide complementary views of codon bias, composition, and folding propensity and are widely used to summarize codon-optimization outcomes.^28^

Across the test set, COformer designs exhibited higher CAI values, higher overall GC and GC3 fractions, and lower uridine fractions than most comparators (Figures 5A–5E). We report uridine fraction as a descriptive feature, as uridine enrichment has been linked to stability- and sensing-related properties in some contexts, without implying direct causality.^19^^,^^57^^,^^58^^,^^59^ Predicted structural tendencies were assessed using ViennaRNA. COformer-generated sequences showed MFE values in a range comparable to other methods (Figure 5F), indicating that the observed codon-usage and composition shifts were not accompanied by extreme changes in predicted folding propensity. Codon pair bias (CPB) distributions were additionally examined as a descriptive measure of local codon-pair composition, revealing method-dependent differences across the held-out test set (Figure S5). To further examine whether COformer collapses toward a single preferred codon within each amino acid family, we computed the per-amino-acid top-codon ratio and compared COformer with a transformer baseline trained under identical conditions. COformer showed reduced dominance for multiple amino acids, indicating more diverse synonymous codon selection within the evaluated benchmark (Figure S6).

Together, these results position COformer within the spectrum of existing commercial and learning-based approaches under identical evaluation conditions and show that COformer generates sequences with codon-usage and composition profiles consistent with commonly used optimization metrics while maintaining broadly comparable predicted structural characteristics.

## Discussion

Codon optimization is a key strategy for enhancing protein expression, traditionally guided by codon frequency tables or codon adaptation indices such as CAI.^6^^,^^20^ While effective in many settings, these approaches often overlook local codon dependencies and the influence of long-range sequence context, both of which can influence expression-related sequence properties. COformer was designed to address these limitations by integrating convolutional layers that capture short-range codon patterns with transformer encoders that model long-range dependencies across the coding sequence. In addition, contrastive learning was employed to encourage consistency between amino acid and synonymous codon representations beyond simple frequency-based encoding. Trained on *Homo sapiens* sequences optimized by widely used commercial tools, COformer learns recurrent codon-selection patterns observed across heterogeneous optimization strategies used in practice, rather than assuming a single universally optimal synonymous solution.

Importantly, codon optimization does not have a single experimentally defined ground-truth sequence for each protein. Because most amino acids can be encoded by multiple synonymous codons, the synonymous design space is highly non-unique, and codon preferences depend on host, transcript, cellular, and application-specific constraints.^6^^,^^31^^,^^33^^,^^34^ In addition, transcript architecture and RNA-processing context may influence how coding-sequence features are coupled to RNA maturation, export, stability, and translation.^18^^,^^33^ Consequently, existing optimization tools encode heuristic and context-specific design choices rather than a single universally valid principle of codon selection. COformer explicitly operates within this paradigm: it does not assume a universal or gene-invariant rule for synonymous codon usage, but instead learns reproducible statistical regularities from sequences generated by optimization strategies widely used in practical gene design. Moreover, the optimal design principles for codon optimization may vary depending on the intended application. For example, RNA vaccines delivered directly to the cytoplasm may primarily benefit from enhanced translational efficiency and balanced structural stability, whereas endogenous or nucleus-exported transcripts may additionally depend on RNA processing and nuclear export dynamics.^11^^,^^12^^,^^17^

Notably, pairwise similarity analyses revealed substantial divergence among commercially optimized sequences despite identical protein inputs, indicating that codon optimization does not converge to a single synonymous solution space. This non-uniqueness was further reflected experimentally, as the ExpOptimizer-optimized EMG1 construct exhibited lower expression than the corresponding wild-type sequence despite being optimized according to the tool’s design strategy. These observations highlight the context-dependent limitations of heuristic optimization strategies and suggest that improvements in individual sequence indices do not necessarily translate into consistent expression outcomes across genes. In contrast, COformer showed consistently higher expression across the three tested genes, suggesting that learning contextual codon-selection tendencies from heterogeneous optimization landscapes may help improve robustness across sequence contexts. Rather than converging toward a single codon-usage heuristic, exposure to heterogeneous optimization strategies may enable the model to learn broader contextual regularities that remain reproducible across distinct sequence environments.

Across *in silico* analyses and HEK293T cell experiments, COformer generated coding sequences with improvements across several sequence-level metrics, including CAI, tAI, GC content, and MFE, relative to both wild-type and conventionally optimized references. Experimental evaluation using *EMG1*, *JNK1*, and *CREB1* further demonstrated enhanced protein expression. RNA-seq analysis also revealed differences in steady-state transcript abundance among synonymous variants, suggesting that transcript-level effects may partially contribute to the observed protein-output gains. These observations further support the view that codon optimization cannot be fully explained by isolated scalar metrics alone, but instead reflects interactions among translational, structural, and transcript-level regulatory processes. A stability-focused variant of the model achieved additional gains in MFE and GC content without compromising codon adaptation, highlighting the flexibility of the framework and its relevance to applications where structural robustness is prioritized, such as gene therapy.

Nevertheless, several limitations of the present study warrant careful consideration. The training data were restricted to human-derived coding sequences, and experimental validation was conducted in a single cell line, which constrains direct generalization across species and cellular contexts. Residual variation in GC content persists despite multi-objective optimization, and reliance on commercially optimized sequences may introduce biases specific to the underlying design heuristics of those tools. In addition, the present framework does not explicitly model factors such as tRNA modifications, ribosome dynamics, nuclear export efficiency, or transcript-specific post-transcriptional regulation, which may further influence codon-dependent expression outcomes.^32^ More broadly, any learned optimization strategy should be interpreted as contextually effective rather than universally optimal, and the relative importance of these factors may differ depending on the intended application and transcript context.^34^

From a translational perspective, AI-based codon optimization has potential implications for improving gene therapy vector design. In hematopoietic stem and progenitor cells, for example, commonly used viral promoters such as CMV are rapidly silenced by epigenetic mechanisms, limiting long-term transgene expression.^66^ By tuning expression at the coding sequence level, COformer offers an orthogonal strategy that complements promoter and enhancer engineering. However, extending codon optimization to therapeutic settings requires accounting for additional sequence-level constraints. Synonymous substitutions can inadvertently introduce cryptic splice donor or acceptor motifs within coding regions, leading to aberrant splicing and loss of therapeutic efficacy.^7^ Likewise, 5′ and 3′ untranslated regions (UTRs) play critical roles in mRNA stability, translation initiation, and tissue specificity and represent natural targets for joint optimization.^13^^,^^56^ Furthermore, as tRNA abundance and codon usage preferences vary across tissues, incorporating cell-type-specific constraints will be essential for improving both safety and durability of expression.^30^

Future work will focus on expanding training datasets to encompass diverse host species and cellular contexts, integrating UTR features and splicing-aware constraints, and exploring multi-objective or reinforcement learning strategies to jointly optimize expression, stability, and manufacturability. By leveraging empirical regularities in codon usage, COformer provides a flexible, data-driven framework for context-aware synthetic gene design in mammalian expression systems.

## Materials and methods

### Dataset construction

We retrieved 18,467 reviewed *Homo sapiens* protein sequences from UniProt to ensure curated annotations.^67^ For each protein, we generated three synonymous coding-sequence variants using commercially available codon-optimization tools—ExpOptimizer (NovoPro), GenSmart (GenScript), and GeneArt (Thermo Fisher Scientific)—under each tool’s default settings for human expression, unless otherwise stated.^38^^,^^39^^,^^40^ This yielded 55,401 optimized coding sequences (three variants per protein). Because codon optimization does not yield a single ground-truth target sequence, these tool-generated sequences were used as proxy design targets to expose COformer to diverse heuristic codon-selection regimes.

All generated coding sequences were verified to be in-frame and to encode the intended amino acid sequence under the standard genetic code. Nucleotide representations (DNA/RNA alphabet) were handled consistently across methods to ensure comparability of downstream sequence-level analyses. To prevent information leakage, all sequences derived from the same protein (the three tool-optimized variants) were assigned to the same dataset split (training, validation, or test), so evaluation reflects generalization to unseen proteins rather than protein-specific memorization.

### Model architecture and training strategy

**Figure 6:**
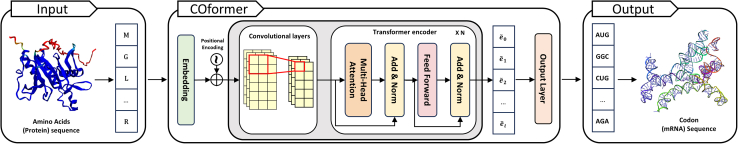
COformer architecture and inference pipeline During inference, COformer receives an amino acid sequence as input and predicts codon-token probabilities at each position. The input sequence is embedded and processed by a convolutional module, followed by transformer encoder layers and a position-wise output head. During decoding, non-synonymous codons are masked so that each selected codon encodes the corresponding input amino acid.

We developed COformer, which integrates convolutional layers with transformer encoder layers to learn short-range and long-range context in protein-coding sequences. An overview of the framework, from amino acid input to optimized codon sequence output, is shown in Figure 6. During inference, COformer takes an amino acid token sequence as input; codon-token sequences are used only during training for auxiliary supervision and contrastive regularization.

Let $x^{aa}=\left[x_{0}^{aa},x_{1}^{aa},\ldots ,x_{n-1}^{aa}\right]$ denote an amino acid token sequence of length *n*, where $x_{i}^{aa}$ is a discrete token index. An embedding layer maps **x**^*aa*^ to $E_{0}\in R^{n\times d}$, where *d* is the embedding dimensionality:

(Equation 1) $$E_{0}=\text{EmbeddingLayer}\left(x^{aa}\right)\text{,}$$

To summarize local sequence context, we apply a 1D convolution with kernel size *k* and stride *s*:

(Equation 2) $$H=\text{Conv}1D\left(E_{0};W_{\text{conv}},b_{\text{conv}}\right)\text{,}$$

followed by a nonlinearity and batch normalization,

(Equation 3) $$\tilde{H}=\text{BN}\left(\sigma \left(H\right)\right)\text{,}$$

A residual connection is used to preserve the original embedding information:

(Equation 4) $$E_{\text{conv}}=\tilde{H}+\text{Proj}\left(E_{0}\right)\text{,}$$

where Proj(·) denotes an identity mapping when dimensions match or a linear projection when dimensions differ. When pooling is used in the implementation, it is applied in a length-preserving manner, or followed by a corresponding projection, to maintain compatibility with the residual pathway. This convolutional representation provides locally contextualized embeddings that are subsequently processed by the transformer encoders.

After local features are captured, **E**_conv_ is passed through multiple transformer encoder layers, each consisting of a multi-head self-attention module and a position-wise feedforward network:

(Equation 5) $${\tilde{E}}^{aa}=\text{Encoders}\left(E_{\text{conv}}\right)$$

The final encoder output ${\tilde{E}}^{aa}$ is mapped to codon-selection logits using a position-wise prediction head, and a softmax yields position-wise probability distributions over the codon vocabulary:

(Equation 6) $${\hat{y}}^{aa}=\text{Softmax}\left(\text{OutputHead}\left({\tilde{E}}^{aa}\right)\right)$$

Here, OutputHead(·) denotes a position-wise classifier that maps each contextualized embedding to logits over the codon vocabulary. During decoding, logits for non-synonymous codons are masked so that codon selection at each position is constrained to synonymous codons for the target amino acid. To quantify codon-choice concentration relative to a transformer baseline trained under identical conditions, we computed a per-amino-acid top-codon ratio on decoded sequences from the held-out test set. Higher values indicate stronger concentration on a single synonymous codon.

**Figure 7:**
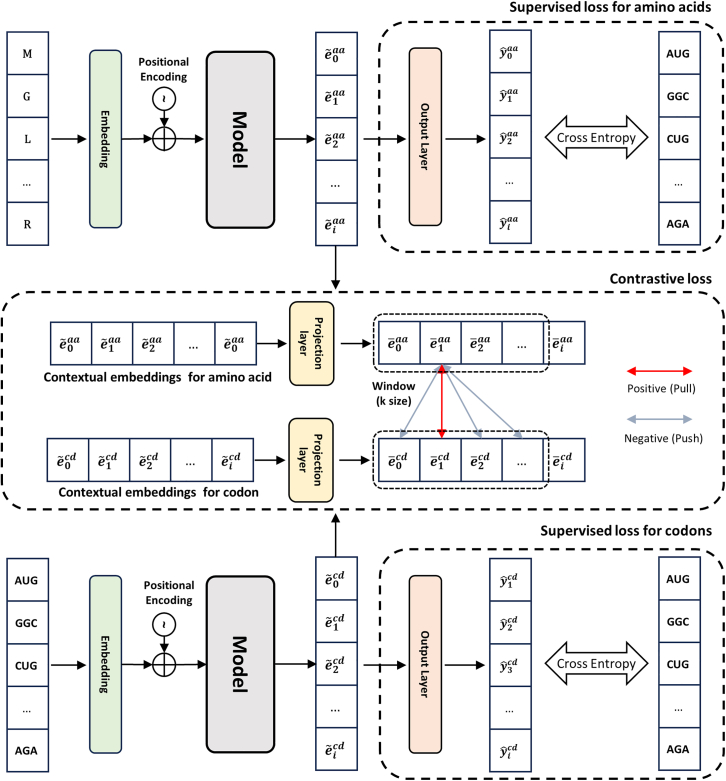
Training objectives used for COformer Schematic overview of the loss function used for COformer training, comprising supervised losses for amino acid and codon-token inputs and a local contrastive term computed between contextualized amino acid and codon representations. For long sequences, the contrastive term was computed over sliding windows and averaged across windows.

An overview of the objectives used to train COformer is shown in Figure 7. Given amino acid inputs **x**^*aa*^ and their corresponding codon-token sequences **x**^*cd*^, COformer computes contextualized representations for the amino acid and codon-token streams using shared encoder layers after stream-specific token embedding, yielding ${\tilde{E}}^{aa}$ and ${\tilde{E}}^{cd}$. These representations are projected into a shared latent space:

(Equation 7) $$z^{aa}=\text{ProjectionLayer}\left({\tilde{E}}^{aa}\right),z^{cd}=\text{ProjectionLayer}\left({\tilde{E}}^{cd}\right)$$

We define an InfoNCE-style contrastive loss using cosine similarity sim(·, ·) and temperature *τ*. For long sequences, we compute the contrastive term over sliding windows of length *L* = 64 tokens with step size *S* = 32 tokens. For sequences shorter than *L*, the full sequence is used as a single window. Let $z_{w}^{aa}=z^{aa}\left[wS:wS+L\right]$ and $z_{w}^{cd}=z^{cd}\left[wS:wS+L\right]$ denote the embeddings restricted to the *w*-th window. Within each window, the matched pair is $\left(z_{w,i}^{aa},z_{w,i}^{cd}\right)$, and codon embeddings at other positions *j* ≠ *i* serve as negatives for $z_{w,i}^{aa}$:

(Equation 8) $$L_{NCE}\left(z_{w}^{aa},z_{w}^{cd}\right)=-\sum_{i=1}^{L}\log\left(\frac{\exp\left(\frac{sim\left(z_{w,i}^{aa},z_{w,i}^{cd}\right)}{\tau }\right)}{\sum_{j=1}^{L}\;\exp\left(\frac{sim\left(z_{w,i}^{aa},z_{w,j}^{cd}\right)}{\tau }\right)}\right)\text{,}$$

The number of windows is W = ⌊(n−L)/S⌋ + 1 for sequences of length *n* ≥ *L*, and the final contrastive loss is averaged over windows:

(Equation 9) $$L_{con}=\frac{1}{W}\sum_{w=0}^{W-1}L_{\text{NCE}}\left(z_{w}^{aa},z_{w}^{cd}\right)$$

During training, COformer is optimized with a primary supervised objective defined on amino acid inputs together with an auxiliary supervised objective on codon-token inputs. Amino acid sequences are mapped to codon-token probabilities ${\hat{y}}^{aa}$, and we minimize cross-entropy with the target codon-token sequence **y**:

(Equation 10) $$L_{aa}=\text{CrossEntropy}\left({\hat{y}}^{aa},y\right)$$

For auxiliary supervision, codon-token inputs are mapped to ${\hat{y}}^{cd}$ with

(Equation 11) $${\hat{y}}^{cd}=\text{Softmax}\left(\text{OutputHead}\left({\tilde{E}}^{cd}\right)\right),L_{cd}=\text{CrossEntropy}\left({\hat{y}}^{cd},y\right)$$

Cross-entropy is computed position-wise over codon tokens and averaged across sequence positions. The supervised loss is

(Equation 12) $$L_{\sup}=L_{aa}+\lambda L_{cd}$$

where *λ* controls the contribution of the auxiliary codon-token supervision term. The overall objective combines supervised and contrastive terms:

(Equation 13) $$L_{total}=L_{\sup}+\mu L_{con}$$

where *μ* balances the supervised and contrastive losses.

### Plasmid construction

The coding sequences of *EMG1*, *JNK1*, and *CREB1* were obtained from the NCBI Gene database.^68^ Codon-optimized coding sequences were designed using NovoPro ExpOptimizer,^38^ GenScript GenSmart,^39^ and COformer. Subsequently, the genes were amplified using KOD-Plus-High Fidelity DNA polymerase following the manufacturer’s protocol (TOYOBO). The wild-type and codon-optimized coding sequences are provided in Tables S1–S3, and primer sequences are provided in Tables S4–S6 (UTR sequences are provided in Table S7). The amplified gene products were then subcloned into the mammalian expression vector pAcGFP1-N1. The constructs were generated using Gibson Assembly Master Mix (New England Biolabs) and verified by DNA sequencing. All constructs contain a C-terminal 6×His-tag.

### Cell culture and transfection of nucleic acids

HEK293T cells were maintained in DMEM (Gibco) supplemented with antibiotics and 10% fetal bovine serum (Gibco) at 37°C in an atmosphere of 5% CO2 in air. The cells (5×10^5^) were plated into 6-well plates. The next day, each plasmid DNA was transfected into monolayer cells as follows. Briefly, each plasmid DNA and P3000 reagent were diluted together in one tube with Opti-MEM medium, while Lipofectamine 3000 reagent (Invitrogen) was diluted separately in another tube with Opti-MEM medium. The contents of both tubes were then gently mixed and incubated for 10 min to form DNA-Lipofectamine complexes. The complexes were added directly to cells in culture medium, and then cells were incubated for 24 h at 37°C prior to experiment.

### Western blot analysis

Cells were lysed in RIPA buffer (Sigma) containing protease inhibitor cocktail (Sigma). The protein concentration of samples was measured using Pierce BCA Protein Assay Kits (Thermo Fisher Scientific). Equal amounts of heat-denatured proteins in sodium dodecyl sulfate (SDS) loading buffer (BIOSESANG) were separated on a 10% SDS-polyacrylamide gel electrophoresis (SDS-PAGE) and transferred to a nitrocellulose membrane using a Mini Trans-Blot Electrophoretic Transfer Cell (Bio-Rad). The membrane was then blocked with a blocking solution (5% non-fat skim milk in Tris-buffered saline with Tween 20 [TBST]) for 1 h. Antibodies against 6×His-tag (1:1,000; Invitrogen, MA1-21315) and GAPDH (1:1,000; Santa Cruz, sc-365062) were incubated with the membrane at 4°C overnight. Following five washes with TBST, the membrane was incubated with HRP-conjugated secondary antibody (1:5,000; Invitrogen, 31430) at room temperature for 1 h. Subsequently, the membrane underwent five washes with TBST. The target bands were visualized by the reaction with enhanced chemiluminescence (ECL) (Thermo Fisher Scientific). Statistical significance among multiple groups was analyzed using one-way ANOVA followed by Tukey’s multiple comparison test. Differences were considered statistically significant at *p* < 0.05.

### Transient transfection of plasmids and cell preparation

Each plasmid (2.5 *μ*g) was transfected into 293T cells in 6-well plates using TransIT-293 transfection reagent (Mirus, #MIR2704) following the manufacturer’s protocol. After 24 h of transfection, medium was removed from the wells, and transfected cells were collected in 1 mL of cold PBS. The cells were washed three times by centrifuging at 400 × g for 5 min in 4°C and resuspending with cold PBS. The final cell pellets were immediately used for RNA extraction.

### RNA extraction and high-throughput sequencing

The RNAs were extracted from cell pellets using Qiagen AllPrep DNA/RNA mini kit (Qiagen #80204). Three micrograms of purified total RNA for each sample was shipped for genome-wide RNA sequencing (Plasmidsaurus).

### RNA-seq data analysis

Single-end RNA-seq reads were quantified using kallisto (v.0.50.1) with a custom reference index. The reference was constructed by combining the cDNA sequences of all 12 overexpression constructs (wild-type, ExpOptimizer, GenSmart, and COformer variants for EMG1, JNK1, and CREB1) together with the human *GAPDH* transcript (ENST00000229239.10) as an internal normalization control. The kallisto index was built with default k-mer length (k = 31), and quantification was performed in single-end mode with an estimated fragment length of 200 bp and standard deviation of 20 bp. To account for cross-mapping artifacts arising from sequence similarity among codon-optimized variants of the same gene, a background subtraction approach was employed. For each gene, the average total mapped counts (summed across all four transcript variants of that gene) from samples overexpressing a different gene were calculated and used as the background estimate. For each overexpression sample, the total counts mapping to all four variants of the target gene were summed to capture reads redistributed by the aligner, and the background estimate was subtracted to obtain the corrected expression value. Corrected counts were then normalized to the *GAPDH* estimated counts from the same sample to control for differences in library size, RNA yield, and transfection efficiency. Final expression values were reported as fold-change relative to the wild-type construct for each gene.

## Data and code availability

All sequence information generated in this study is provided in the Supplementary Tables. Tables S1–S3 list the wild-type and codon-optimized sequences used for expression analysis of *EMG1*, *JNK1*, and *CREB1*, respectively. Tables S4–S6 contain the primer sequences used to amplify each gene, and Table S7 provides the 5′ and 3′ UTR sequences used in the constructs.

• A total of 18,467 reviewed Swiss-Prot protein sequences from *Homo sapiens* were obtained from the UniProt database (https://uniprot.org) and used for model training and evaluation.
• The codebase used for model training, evaluation, and sequence analysis in this study is publicly available at https://github.com/PNUMLB/COformer.
• The COformer web tool is publicly available at http://mlblabcoformer.org.

## Acknowledgments

This work was supported by the 10.13039/501100003725National Research Foundation of Korea (NRF) (RS-2023-00257479) and the Institute of Information & Communications Technology Planning & Evaluation (IITP) through the Artificial Intelligence Convergence Innovation Human Resources Development program (IITP-2026-RS-2023-00254177), both funded by the Korean government (MSIT). This research was also supported by the Regional Innovation System & Education (RISE) program through the Institute for Regional Innovation System & Education in Busan Metropolitan City, funded by the 10.13039/501100002701Ministry of Education (MOE) and the 10.13039/501100009819Busan Metropolitan City, Republic of Korea (2026-RISE-02-004). These grants were awarded to G.S.

## Author contributions

Juseong Kim and Jeongmu Kim designed the machine learning approaches, developed the tools, collected the data, performed the analysis, and wrote the paper. J.-W.L. designed and performed the protein expression experiments and wrote the paper. Q.Q. and C.D. performed the transient transfection, RNA extraction, and RNA-seq experiments. H.Y.K. and G.S. initiated the research. Y.C. and H.Y.K. contributed to writing the paper. G.S. supervised, coordinated the work, and wrote the paper. All authors reviewed and approved the final manuscript.

## Declaration of interests

The authors declare no competing interests.

## Declaration of generative AI and AI-assisted technologies in the writing process

During the preparation of this work, the authors used ChatGPT in order to improve readability and language. After using this tool/service, the authors reviewed and edited the content as needed and take full responsibility for the content of the publication.

## Supplemental information

Document S1. Figures S1–S6 and Tables S1–S7

Document S2. Article plus supplemental information

## References

[1] Hacein-Bey-Abina S., Hauer J., Lim A., Picard C., Wang G.P., Berry C.C., Martinache C., Rieux-Laucat F., Latour S., Belohradsky B.H. (2010). Efficacy of gene therapy for X-linked severe combined immunodeficiency. N. Engl. J. Med..

[2] Varma N., Janic B., Ali M., Iskander A., Arbab A. (2011). Lentiviral based gene transduction and promoter studies in human hematopoietic stem cells (hHSCs). J. Stem Cells Regen. Med..

[3] Montiel-Equihua C.A., Zhang L., Knight S., Saadeh H., Scholz S., Carmo M., Alonso-Ferrero M.E., Blundell M.P., Monkeviciute A., Schulz R. (2012). The β-globin locus control region in combination with the EF1α short promoter allows enhanced lentiviral vector-mediated erythroid gene expression with conserved multilineage activity. Mol. Ther..

[4] Weissman D., Karikó K. (2015). mRNA: fulfilling the promise of gene therapy. Mol. Ther..

[5] Kitada T., DiAndreth B., Teague B., Weiss R. (2018). Programming gene and engineered-cell therapies with synthetic biology. Science.

[6] Mauro V.P., Chappell S.A. (2014). A critical analysis of codon optimization in human therapeutics. Trends Mol. Med..

[7] Lin B.C., Kaissarian N.M., Kimchi-Sarfaty C. (2023). Implementing computational methods in tandem with synonymous gene recoding for therapeutic development. Trends Pharmacol. Sci..

[8] Kudla G., Murray A.W., Tollervey D., Plotkin J.B. (2009). Coding-sequence determinants of gene expression in Escherichia coli. Science.

[9] Mauro V.P. (2018). Codon optimization in the production of recombinant biotherapeutics: potential risks and considerations. BioDrugs.

[10] Zhang H., Zhang L., Lin A., Xu C., Li Z., Liu K., Liu B., Ma X., Zhao F., Jiang H. (2023). Algorithm for optimized mRNA design improves stability and immunogenicity. Nature.

[11] Kowalski P.S., Rudra A., Miao L., Anderson D.G. (2019). Delivering the messenger: advances in technologies for therapeutic mRNA delivery. Mol. Ther..

[12] Sahin U., Karikó K., Türeci Ö. (2014). mRNA-based therapeutics—developing a new class of drugs. Nat. Rev. Drug Discov..

[13] Orlandini von Niessen A.G., Poleganov M.A., Rechner C., Plaschke A., Kranz L.M., Fesser S., Diken M., Löwer M., Vallazza B., Beissert T. (2019). Improving mRNA-based therapeutic gene delivery by expression-augmenting 3′ UTRs identified by cellular library screening. Mol. Ther..

[14] Hanson G., Coller J. (2018). Codon optimality, bias and usage in translation and mRNA decay. Nat. Rev. Mol. Cell Biol..

[15] Brule C.E., Grayhack E.J. (2017). Synonymous codons: choose wisely for expression. Trends Genet..

[16] Boël G., Letso R., Neely H., Price W.N., Wong K.H., Su M., Luff J., Valecha M., Everett J.K., Acton T.B. (2016). Codon influence on protein expression in E. coli correlates with mRNA levels. Nature.

[17] Pardi N., Hogan M.J., Porter F.W., Weissman D. (2018). mRNA vaccines—a new era in vaccinology. Nat. Rev. Drug Discov..

[18] Radrizzani S., Kudla G., Izsvák Z., Hurst L.D. (2024). Selection on synonymous sites: the unwanted transcript hypothesis. Nat. Rev. Genet..

[19] Presnyak V., Alhusaini N., Chen Y.H., Martin S., Morris N., Kline N., Olson S., Weinberg D., Baker K.E., Graveley B.R., Coller J. (2015). Codon optimality is a major determinant of mRNA stability. Cell.

[20] Sharp P.M., Li W.H. (1986). An evolutionary perspective on synonymous codon usage in unicellular organisms. J. Mol. Evol..

[21] Zuker M., Stiegler P. (1981). Optimal computer folding of large RNA sequences using thermodynamics and auxiliary information. Nucleic Acids Res..

[22] Gruber A.R., Bernhart S.H., Lorenz R. (2015). The ViennaRNA web services. Methods Mol. Biol..

[23] Wu Q., Medina S.G., Kushawah G., DeVore M.L., Castellano L.A., Hand J.M., Wright M., Bazzini A.A. (2019). Translation affects mRNA stability in a codon-dependent manner in human cells. eLife.

[24] Hoekema A., Kastelein R.A., Vasser M., de Boer H.A. (1987). Codon replacement in the PGK1 gene of Saccharomyces cerevisiae: experimental approach to study the role of biased codon usage in gene expression. Mol. Cell Biol..

[25] Andersson S.G., Kurland C.G. (1990). Codon preferences in free-living microorganisms. Microbiol. Rev..

[26] Yang K.K., Wu Z., Arnold F.H. (2019). Machine-learning-guided directed evolution for protein engineering.Nature. Methods.

[27] Castillo-Hair S.M., Seelig G. (2022). Machine learning for designing next generation mRNA therapeutics. Acc. Chem. Res..

[28] Fu H., Liang Y., Zhong X., Pan Z., Huang L., Zhang H., Xu Y., Zhou W., Liu Z. (2020). Codon optimization with deep learning to enhance protein expression. Sci. Rep..

[29] Jain R., Jain A., Mauro E., LeShane K., Densmore D. (2023). ICOR: improving codon optimization with recurrent neural networks. BMC Bioinf..

[30] Ravi S., Sharma T., Yip M., Yang H., Xie J., Gao G., Tai P.W.L. (2025). A deep learning model trained on expressed transcripts across different tissue types reveals cell-type codon-optimization preferences. Nucleic Acids Res..

[31] Fallahpour A., Gureghian V., Filion G.J., Lindner A.B., Pandi A. (2025). CodonTransformer: a multispecies codon optimizer using context-aware neural networks. Nat. Commun..

[32] Quax T.E.F., Claassens N.J., Söll D., van der Oost J. (2015). Codon bias as a means to fine-tune gene expression. Mol. Cell.

[33] Plotkin J.B., Kudla G. (2011). Synonymous but not the same: the causes and consequences of codon bias. Nat. Rev. Genet..

[34] Paremskaia A.I., Kogan A.A., Murashkina A., Naumova D.A., Satish A., Abramov I.S., Feoktistova S.G., Mityaeva O.N., Deviatkin A.A., Volchkov P.Y. (2024). Codon-optimization in gene therapy: promises, prospects and challenges. Front. Bioeng. Biotechnol..

[35] Tuller T., Carmi A., Vestsigian K., Navon S., Dorfan Y., Zaborske J., Pan T., Dahan O., Furman I., Pilpel Y. (2010). An evolutionarily conserved mechanism for controlling the efficiency of protein translation. Cell.

[36] van den Oord A., Li Y., Vinyals O. (2018). Representation learning with contrastive predictive coding. arXiv.

[37] Rao R., Meier J., Sercu T., Ovchinnikov S., Rives A. (2020). Transformer protein language models are unsupervised structure learners. bioRxiv.

[38] NovoPro ExpOptimizer codon optimization tool. https://www.novoprolabs.com/tools/codon-optimization.

[39] GenScript GenSmart codon optimization tool. https://www.genscript.com/tools/gensmart-codon-optimization.

[40] Thermo Fisher Scientific GeneArt codon optimization tool. https://www.thermofisher.com/order/geneart-dashboard/index.html.

[41] dos Reis M., Savva R., Wernisch L. (2004). Solving the riddle of codon usage preferences: a test for translational selection. Nucleic Acids Res..

[42] Fath S., Bauer A.P., Liss M., Spriestersbach A., Maertens B., Hahn P., Ludwig C., Schäfer F., Graf M., Wagner R. (2011). Multiparameter RNA and codon optimization: a standardized tool to assess and enhance autologous mammalian gene expression. PLoS One.

[43] To K.K.W., Cho W.C.S. (2021). An overview of rational design of mRNA-based therapeutics and vaccines. Expert Opin. Drug Discov..

[44] Meyer B., Wurm J.P., Kötter P., Leisegang M.S., Schilling V., Buchhaupt M., Held M., Bähr U., Karas M., Heckel A. (2011). The Bowen–Conradi syndrome protein Nep1 (Emg1) has a dual role in eukaryotic ribosome biogenesis, as an essential assembly factor and in the methylation of Ψ1191 in yeast 18S rRNA. Nucleic Acids Res..

[45] Davis R.J. (2000). Signal transduction by the JNK group of MAP kinases. Cell.

[46] Lonze B.E., Ginty D.D. (2002). Function and regulation of CREB family transcription factors in the nervous system. Neuron.

[47] Bray N.L., Pimentel H., Melsted P., Pachter L. (2016). Near-optimal probabilistic RNA-seq quantification. Nat. Biotechnol..

[48] Wang Y., Li Z., Wang X., Jiang W., Jiang W. (2023). SARS-CoV-2 continuously optimizes its codon usage to adapt to human lung environment. J. Appl. Genet..

[49] Ranaghan M.J., Li J.J., Laprise D.M., Garvie C.W. (2021). Assessing optimal: inequalities in codon optimization algorithms. BMC Biol..

[50] Rodriguez A., Wright G., Emrich S., Clark P.L. (2018). %MinMax: A versatile tool for calculating and comparing synonymous codon usage and its impact on protein folding. Protein Sci..

[51] Fox J.M., Erill I. (2010). Relative codon adaptation: a generic codon bias index for prediction of gene expression. DNA Res..

[52] Ikemura T. (1981). Correlation between the abundance of Escherichia coli transfer RNAs and the occurrence of the respective codons in its protein genes: a proposal for a synonymous codon choice that is optimal for the E. coli translational system. J. Mol. Biol..

[53] Bennetzen J.L., Hall B.D. (1982). Codon selection in yeast. J. Biol. Chem..

[54] Gouy M., Gautier C. (1982). Codon usage in bacteria: correlation with gene expressivity. Nucleic Acids Res..

[55] Supek F., Vlahoviček K. (2005). Comparison of codon usage measures and their applicability in prediction of microbial gene expressivity. BMC Bioinf..

[56] Mauger D.M., Cabral B.J., Presnyak V., Su S.V., Reid D.W., Goodman B., Link K., Khatwani N., Reynders J., Moore M.J., McFadyen I.J. (2019). mRNA structure regulates protein expression through changes in functional half-life. Proc. Natl. Acad. Sci. USA.

[57] Karikó K., Buckstein M., Ni H., Weissman D. (2005). Suppression of RNA recognition by Toll-like receptors: the impact of nucleoside modification and the evolutionary origin of RNA. Immunity.

[58] Nance K.D., Meier J.L. (2021). Modifications in an emergency: the role of N1-methylpseudouridine in COVID-19 vaccines. ACS Cent. Sci..

[59] Andries O., Mc Cafferty S., De Smedt S.C., Weiss R., Sanders N.N., Kitada T. (2015). N1-methylpseudouridine-incorporated mRNA outperforms pseudouridine-incorporated mRNA by providing enhanced protein expression and reduced immunogenicity in mammalian cell lines and mice. J. Control. Release.

[60] Cohen N., Dagan T., Stone L., Graur D. (2005). GC composition of the human genome: in search of isochores. Mol. Biol. Evol..

[61] Tatarinova T.V., Alexandrov N.N., Bouck J.B., Feldmann K.A. (2010). GC3 biology in corn, rice, sorghum and other grasses. BMC Genom..

[62] McCaskill J.S. (1990). The equilibrium partition function and base pair binding probabilities for RNA secondary structure. Biopolymers.

[63] Shabalina S.A., Ogurtsov A.Y., Spiridonov N.A. (2006). A periodic pattern of mRNA secondary structure created by the genetic code. Nucleic Acids Res..

[64] Courel M., Clément Y., Bossevain C., Foretek D., Vidal Cruchez O., Yi Z., Bénard M., Benassy M.N., Kress M., Vindry C. (2019). GC content shapes mRNA storage and decay in human cells. eLife.

[65] Qiu Y., Kang Y.M., Korfmann C., Pouyet F., Eckford A., Palazzo A.F. (2024). The GC-content at the 5′ ends of human protein-coding genes is undergoing mutational decay. Genome Biol..

[66] Ngai S.C., Rosli R., Al Abbar A., Abdullah S. (2015). DNA methylation and histone modifications are the molecular lock in lentivirally transduced hematopoietic progenitor cells. Biomed Res. Int..

[67] UniProt Consortium UniProt protein database. https://www.uniprot.org/.

[68] Brown G.R., Hem V., Katz K.S., Ovetsky M., Wallin C., Ermolaeva O., Tolstoy I., Tatusova T., Pruitt K.D., Maglott D.R., Murphy T.D. (2015). Gene: a gene-centered information resource at NCBI. Nucleic Acids Res..

