# Supplementary material for: Enhancing protein expression in humans through codon optimization with transformer and contrastive learning
Source: Mol Ther Nucleic Acids. 2026 Jun 18;37(3):102991. doi: 10.1016/j.omtn.2026.102991 (PMC13330525; doi:10.1016/j.omtn.2026.102991)
Supplement: Document S1. Figures S1–S6 and Tables S1–S7 [file mmc1.pdf]

OMTN, Volume *37*

Supplemental information

Enhancing protein expression in humans through codon optimization with transformer and contrastive learning

Juseong Kim, Jeongmu Kim, Jae-Wook Lee, Qian Qi, Caroline Danehy, Ho Young Kang, Yong Cheng, and Giltae Song

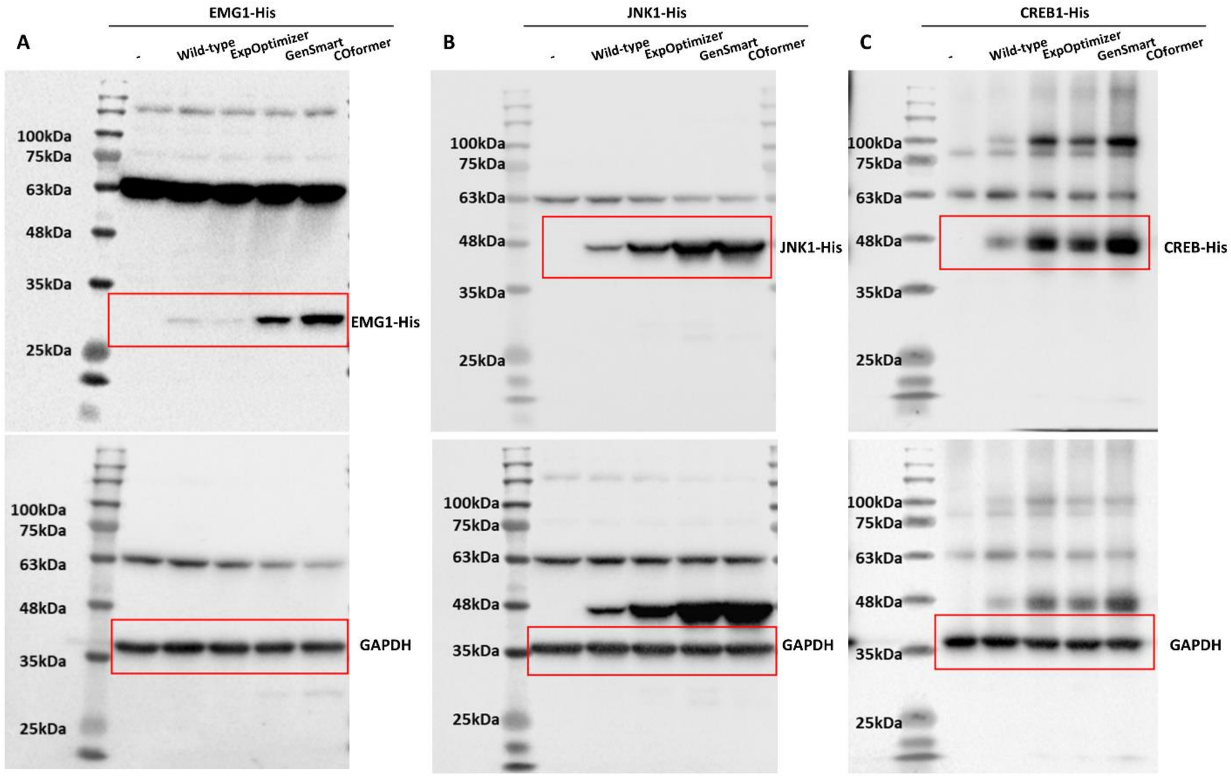

Figure S1. Original western blot images corresponding to Figure 2A. (A–C) Blots showing His-tagged expression of EMG1, JNK1, and CREB1. (D–F) Blots showing GAPDH controls for EMG1, JNK1, and CREB1.

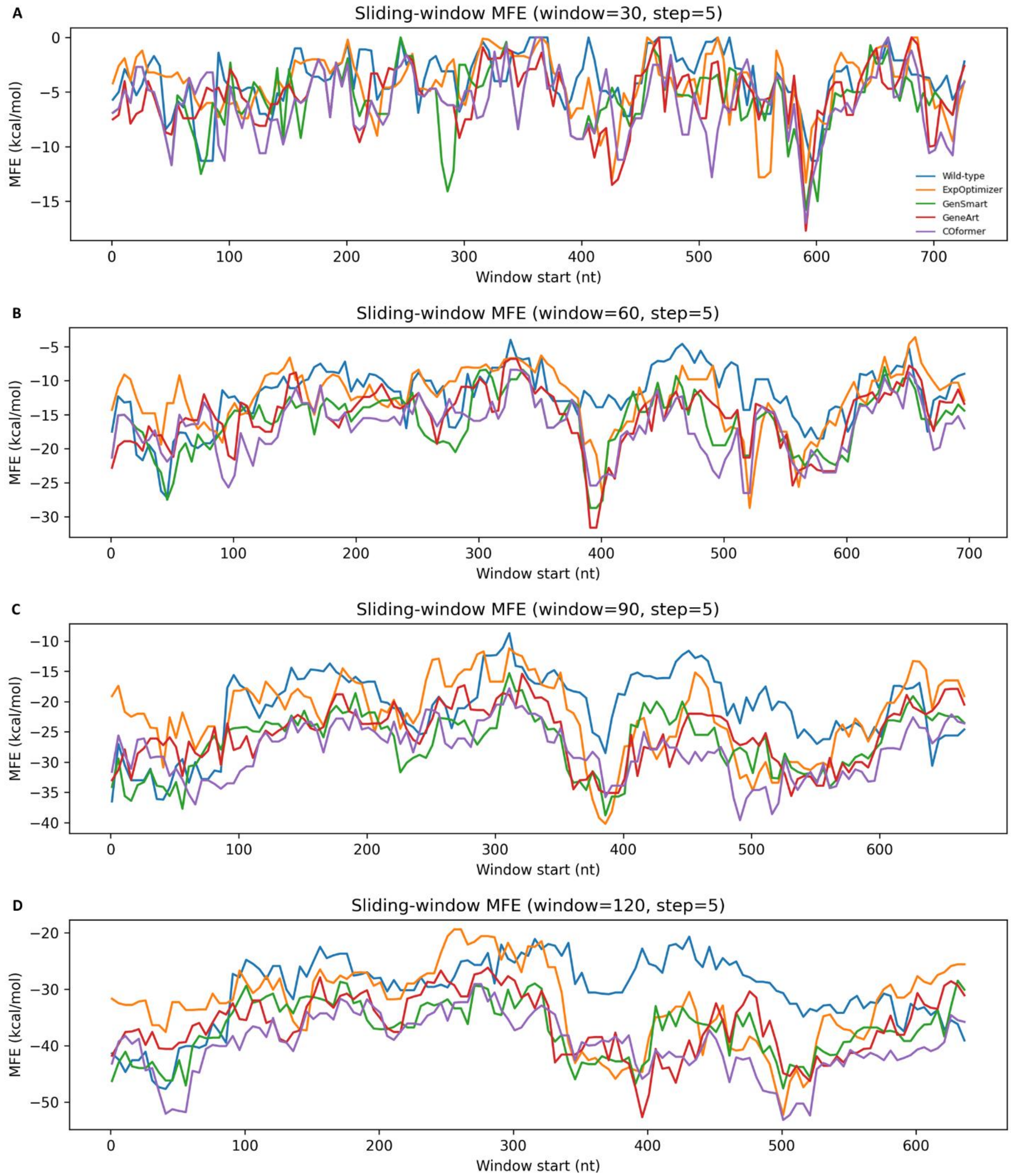

Figure S2. Sliding-window MFE profiles for EMG1. Sliding-window minimum free energy (MFE) was calculated along the coding sequence of EMG1 starting from the start codon using ViennaRNA for the wild-type sequence and sequences optimized by ExpOptimizer, GenSmart, GeneArt, and COformer. Panels (A–D) show overlaid MFE tracks obtained with window sizes of 30, 60, 90, and 120 nt, respectively (step = 5 nt), enabling comparison of local structural stability patterns across optimization methods.

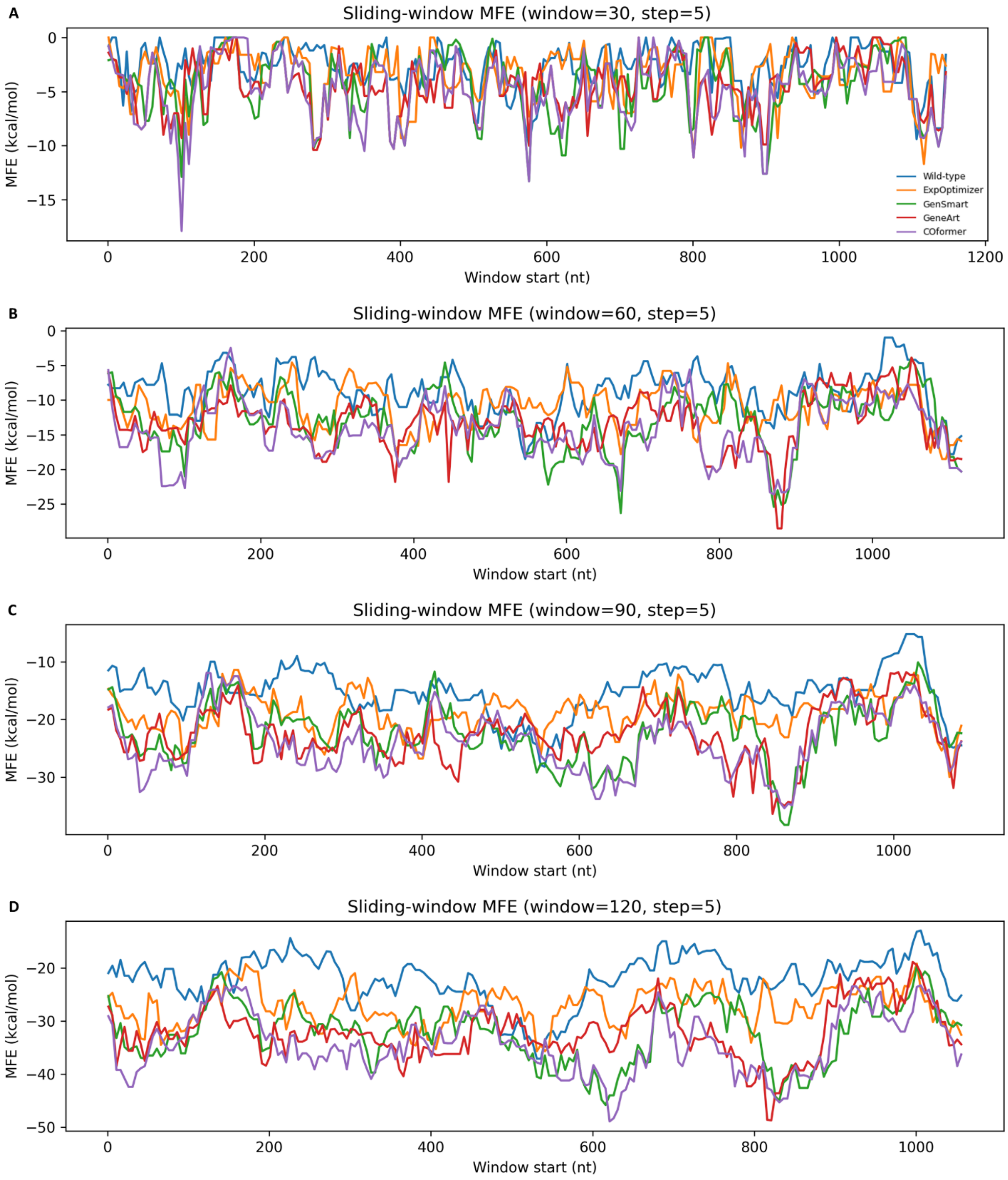

Figure S3. Sliding-window MFE profiles for JNK1. Sliding-window minimum free energy (MFE) was calculated along the coding sequence of JNK1 starting from the start codon using ViennaRNA for the wild-type sequence and sequences optimized by ExpOptimizer, GenSmart, GeneArt, and COformer. Panels (A–D) show overlaid MFE tracks obtained with window sizes of 30, 60, 90, and 120 nt, respectively (step = 5 nt), enabling comparison of local structural stability patterns across optimization methods.

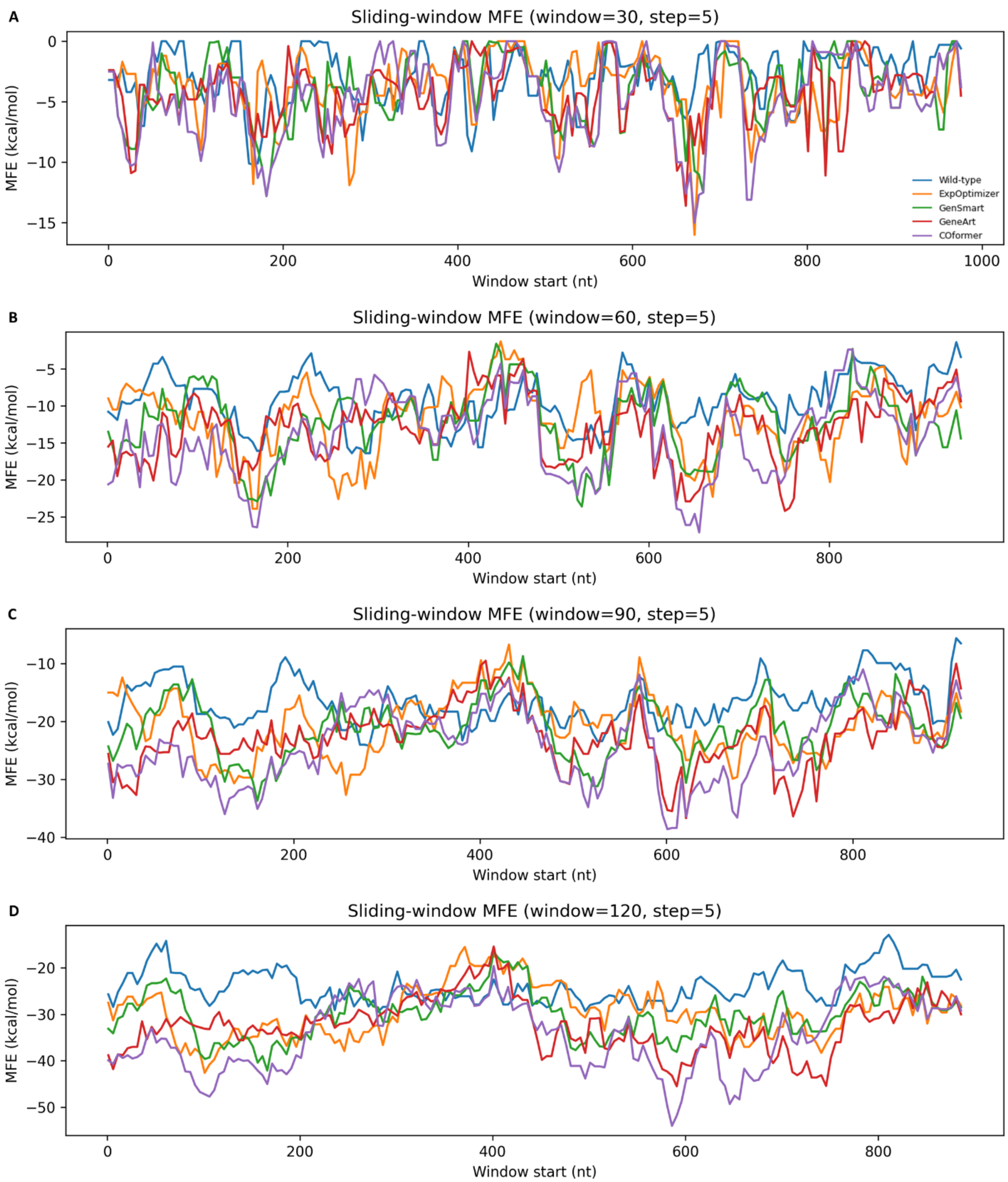

Figure S4. Sliding-window MFE profiles for CREB1. Sliding-window minimum free energy (MFE) was calculated along the coding sequence of CREB1 starting from the start codon using ViennaRNA for the wild-type sequence and sequences optimized by ExpOptimizer, GenSmart, GeneArt, and COformer. Panels (A–D) show overlaid MFE tracks obtained with window sizes of 30, 60, 90, and 120 nt, respectively (step = 5 nt), enabling comparison of local structural stability patterns across optimization methods.

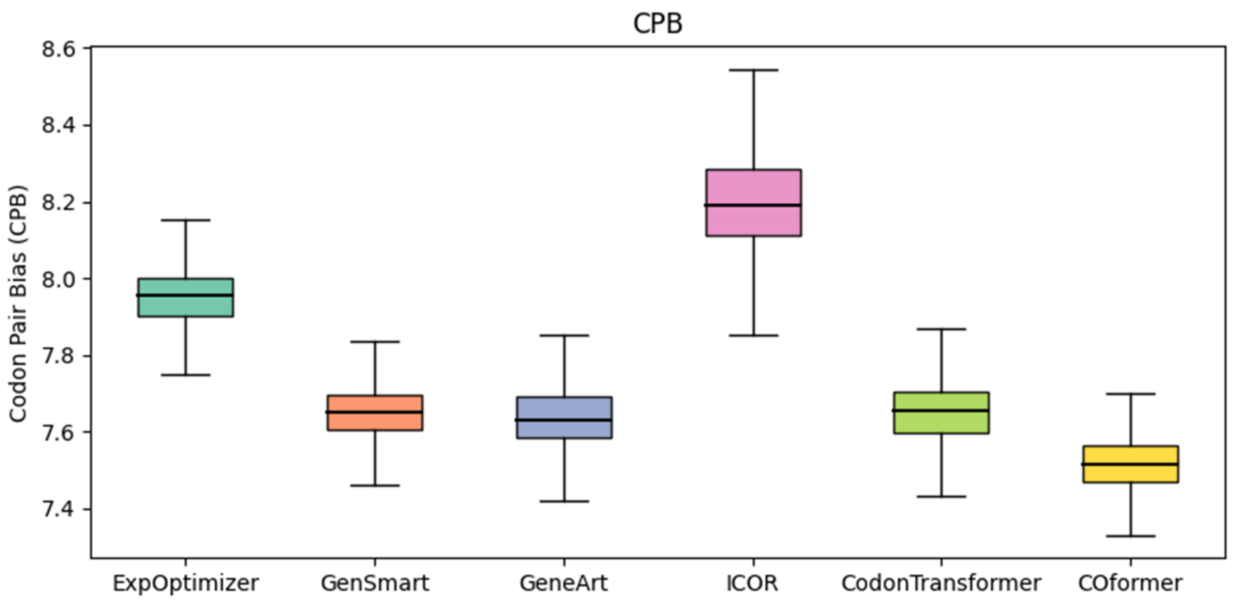

Figure S5. Codon pair bias (CPB) across optimization methods. Codon pair bias (CPB) scores were computed for all sequences in the test set and compared across commercial codon optimization tools, deep learning–based models, and COformer. CPB values are shown as box plots, summarizing the distribution of codon-pair composition for each method.

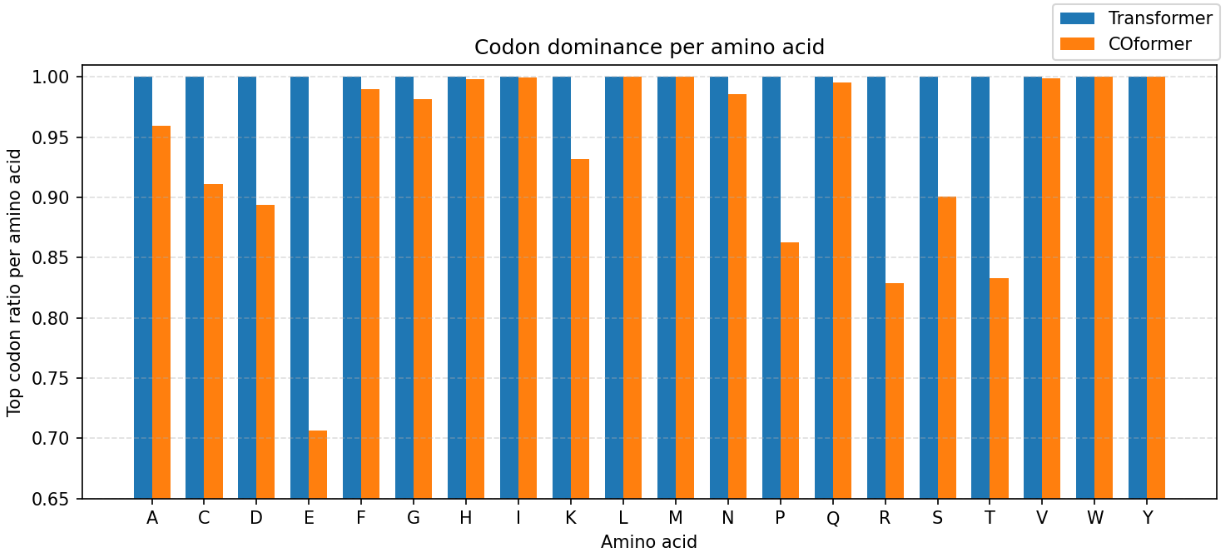

Figure S6. Synonymous codon dominance per amino acid in a transformer baseline versus COformer. To quantify concentration of codon choice within amino-acid families, we computed the top-codon ratio for each amino acid, defined as the fraction of output codons that correspond to the most frequently selected synonymous codon for that amino acid across the held-out test set. Higher values correspond to stronger concentration on a single codon. Under identical training conditions, the transformer baseline shows higher dominance for multiple amino acids, whereas COformer exhibits reduced dominance, reflecting more diverse synonymous codon selection in this benchmark.

Table S1: Sequences of methods for EMG1

Method	Sequence
**Wild-type**	ATGGCCGCGCCCAGTGATGGATTCAAGCCTCGTGAACGAAGCGGTGGGGAGCAGGCACAG<br>GACTGGGATGCTCTGCCACCCAAGCGGCCCCGACTAGGGGCAGGAAACAAGATCGGAGGC<br>CGTAGGCTTATTGTGGTGCTGGAAGGGGCCAGTCTGGAGACAGTCAAGGTAGGGAAGACA<br>TATGAGCTACTCAACTGTGACAAGCACAAGTCTATATTGTTGAAGAATGGACGGGACCCT<br>GGGGAAGCGCGGCCAGATATCACCCACCAGAGTTTGCTGATGCTGATGGATAGTCCCCTG<br>AACCGAGCTGGCTTGCTACAGGTTTATATCCATACACAGAAGAATGTTCTGATTGAAGTG<br>AATCCCCAGACCCGAATTCCCAGAACCTTTGACCGCTTTTGTGGCCTCATGGTTCAACTT<br>TTACACAAGCTCAGTGTTCGAGCAGCTGATGGCCCCCAGAAGCTTTTGAAGGTAATTAAG<br>AATCCAGTATCAGATCACTTTCCAGTTGGATGTATGAAAGTTGGCACTTCTTTTTCCATC<br>CCGGTTGTCAGTGATGTGCGTGAGCTGGTGCCCAGCAGTGATCCTATTGTTTTTGTGGTA<br>GGGGCCTTTGCCCATGGCAAGGTCAGTGTGGAGTATACAGAGAAGATGGTGTCCATCAGT<br>AACTACCCCCTTTCTGCTGCCCTCACCTGTGCAAAACTTACCACAGCCTTTGAGGAAGTA<br>TGGGGGGTCATTCACCATCACCATCATCACTGATAA
**ExpOptimizer**	ATGGCTGCCCCATCCGACGGCTTTAAACCGAGAGAAAGAAGTGGTGGAGAGCAAGCACAA<br>GATTGGGACGCTTTGCCACCAAAAAGACCCCGCCTGGGTGCTGGGAACAAGATTGGGGGC<br>CGCAGGCTTATTGTCGTGCTGGAAGGGGCAAGCCTCGAGACTGTTAAGGTGGGGAAAACT<br>TATGAACTGCTGAACTGCGACAAACATAAGTCCATCCTGCTCAAAAATGGGAGAGACCCA<br>GGCGAGGCCAGGCCCGATATCACACACCAGAGCCTCCTTATGCTGATGGACAGCCCATTG<br>AACAGAGCCGGCCTCTTGCAGGTTTACATACACACCCAGAAGAACGTGCTGATCGAAGTG<br>AACCCACAAACAAGGATACCGCGCACATTTGACAGATTCTGTGGCCTGATGGTACAGCTG<br>CTGCATAAGCTCTCTGTGAGAGCAGCCGATGGGCCACAGAAACTGCTCAAGGTCATCAAA<br>AATCCCGTGAGCGATCACTTCCCTGTGGGCTGTATGAAAGTTGGCACTTCATTCTCAATC<br>CCTGTTGTGAGTGACGTGCGAGAGCTGGTGCCCAGCTCTGACCCTATAGTTTTTGTGGTG<br>GGAGCTTTCGCCCACGGCAAAGTGTCAGTGGAATATACCGAAAAGATGGTGAGCATTTCC<br>AACTATCCGCTTTCCGCCGCACTCACATGCGCCAAATTGACAACTGCCTTTGAAGAGGTC<br>TGGGGAGTGATCCACCACCACCATCACCACTGATGA
**GenSmart**	ATGGCCGCCCCTAGCGACGGCTTCAAGCCTAGAGAGAGAAGCGGCGGCGAGCAGGCCCAG<br>GACTGGGACGCCCTGCCCCCCAAGCGGCCTCGGCTGGGCGCTGGCAACAAGATCGGCGGA<br>CGCAGACTGATCGTGGTCCTGGAAGGAGCCTCCCTCGAGACAGTGAAGGTGGGCAAAACC<br>TACGAGCTGCTGAACTGCGACAAGCACAAAAGCATCCTGCTCAAGAACGGCAGAGATCCT<br>GGCGAAGCCAGACCAGATATCACCCACCAGAGCCTGCTGATGCTGATGGACAGCCCTCTG<br>AATAGGGCTGGTCTGCTGCAAGTGTACATCCACACACAGAAAAACGTGCTGATCGAGGTG<br>AACCCTCAGACCAGAATCCCTAGAACCTTCGACAGATTCTGCGGCCTGATGGTGCAGCTG<br>CTGCATAAGCTGAGCGTGCGGGCCGCTGATGGACCTCAGAAGCTGCTGAAGGTGATTAAG<br>AATCCTGTTTCTGACCACTTCCCCGTGGGATGTATGAAAGTGGGCACCAGCTTTAGCATC<br>CCCGTGGTGTCCGATGTGCGGGAACTGGTGCCAAGCAGCGACCCCATCGTTTTCGTGGTG<br>GGCGCCTTTGCCCACGGCAAGGTGTCTGTGGAATACACCGAGAAGATGGTCTCCATCAGC<br>AACTATCCTCTGTCTGCCGCTCTGACCTGCGCCAAGCTGACAACAGCCTTCGAGGAAGTG<br>TGGGGCGTGATCCACCACCACCATCACCACTGATGA
**COformer**	ATGGCCGCCCCTAGCGACGGCTTCAAGCCTAGAGAGAGAAGCGGCGGCGAGCAGGCCCAG<br>GACTGGGACGCCCTGCCTCCTAAGAGACCTAGACTGGGCGCCGGCAACAAGATCGGCGGC<br>AGAAGACTGATCGTGGTGCTGGAGGGCGCCAGCCTGGAGACCGTGAAGGTGGGCAAGACC<br>TACGAGCTGCTGAACTGCGACAAGCACAAGAGCATCCTGCTGAAGAACGGCAGAGACCCT<br>GGCGAGGCCAGACCTGACATCACCCACCAGAGCCTGCTGATGCTGATGGACAGCCCTCTG<br>AACAGAGCCGGCCTGCTGCAGGTGTACATCCACACCCAGAAGAACGTGCTGATCGAGGTG<br>AACCCTCAGACCAGAATCCCTAGAACCTTCGACAGATTCTGCGGCCTGATGGTGCAGCTG<br>CTGCACAAGCTGAGCGTGAGAGCCGCCGACGGCCCTCAGAAGCTGCTGAAGGTGATCAAG<br>AACCCTGTGAGCGACCACTTCCCTGTGGGCTGCATGAAGGTGGGCACCAGCTTCAGCATC<br>CCTGTGGTGAGCGACGTGAGAGAGCTGGTGCCTAGCAGCGACCCTATCGTGTTCGTGGTG<br>GGCGCCTTCGCCCACGGCAAGGTGAGCGTGGAGTACACCGAGAAGATGGTGAGCATCAGC<br>AACTACCCTCTGAGCGCCGCCCTGACCTGCGCCAAGCTGACCACCGCCTTCGAGGAGGTG<br>TGGGGCGTGATCCACCACCACCATCACCACTGATGA

Table S2: Sequences of methods for JNK1

Method	Sequence
**Wild-type**	ATGAGCAGAAGCAAGCGTGACAACAATTTTTATAGTGTAGAGATTGGAGATTCTACATTC<br>ACAGTCCTGAAACGATATCAGAATTTAAAACCTATAGGCTCAGGAGCTCAAGGAATAGTA

	TGCGCAGCTTATGATGCCATTCTTGAAAGAAATGTTGCAATCAAGAAGCTAAGCCGACCA<br>TTTCAGAATCAGACTCATGCCAAGCGGGCCTACAGAGAGCTAGTTCTTATGAAATGTGTT<br>AATCACAAAAATATAATTGGCCTTTTGAATGTTTTCACACCACAGAAATCCCTAGAAGAA<br>TTTCAAGATGTTTACATAGTCATGGAGCTCATGGATGCAAATCTTTGCCAAGTGATTCAG<br>ATGGAGCTAGATCATGAAAGAATGTCCTACCTTCTCTATCAGATGCTGTGTGGAATCAAG<br>CACCTTCATTCTGCTGGAATTATTCATCGGGACTTAAAGCCCAGTAATATAGTAGTAAAA<br>TCTGATTGCACTTTGAAGATTCTTGACTTCGGTCTGGCCAGGACTGCAGGAACGAGTTTT<br>ATGATGACGCCTTATGTAGTGACTCGCTACTACAGAGCACCCGAGGTCATCCTTGGCATG<br>GGCTACAAGGAAAACGTGGATTTATGGTCTGTGGGGTGCATTATGGGAGAAATGGTTTGC<br>CACAAAATCCTCTTTCCAGGAAGGGACTATATTGATCAGTGGAATAAAGTTATTGAACAG<br>CTTGGAACACCATGTCCTGAATTCATGAAGAAACTGCAACCAACAGTAAGGACTTACGTT<br>GAAAACAGACCTAAATATGCTGGATATAGCTTTGAGAAACTCTTCCCTGATGTCCTTTTC<br>CCAGCTGACTCAGAACACAACAAACTTAAAGCCAGTCAGGCAAGGGATTTGTTATCCAAA<br>ATGCTGGTAATAGATGCATCTAAAAGGATCTCTGTAGATGAAGCTCTCCAACACCCGTAC<br>ATCAATGTCTGGTATGATCCTTCTGAAGCAGAAGCTCCACCACCAAAGATCCCTGACAAG<br>CAGTTAGATGAAAGGGAACACACAATAGAAGAGTGGAAAGAATTGATATATAAGGAAGTT<br>ATGGACTTGGAGGAGAGAACCAAGAATGGAGTTATACGGGGGCAGCCCTCTCCTTTAGCA<br>CAGGTGCAGCAGCACCATCACCATCATCACTGATAA
**ExpOptimizer**	ATGAGCAGAAGTAAAAGGGATAACAATTTCTATAGCGTAGAGATCGGCGACTCTACTTTC<br>ACTGTGCTGAAACGCTACCAGAACCTGAAACCAATTGGGAGCGGAGCTCAGGGTATTGTA<br>TGCGCTGCCTACGATGCAATCTTGGAGAGGAACGTGGCCATTAAGAAGCTGAGTCGACCT<br>TTCCAAAACCAGACACATGCTAAAAGGGCTTATAGGGAATTGGTGCTGATGAAGTGCGTG<br>AATCACAAAAATATCATCGGTCTGCTTAACGTGTTCACCCCACAGAAAAGCCTGGAAGAG<br>TTCCAGGATGTCTACATTGTGATGGAACTTATGGACGCGAAACCTCTGCCAGGTTATTCA<br>AATGGAACTGGACCACGAACGCATGAGTTATCTGCTTTATCAAATGCTGTGCGGGATCAA<br>ACACCTGCATTCAGCAGGCATCATCCACAGAGACTTGAAGCCTTCTAACATTGTGGTGAA<br>GTCAGACTGCACTCTGAAGATTCTGGATTTCGGTCTCGCTCGGACTGCTGGCACCTCATT<br>CATGATGACACCCTACGTCGTTACACGCTACTATAGGGCTCCCGAAGTGATCTTGGGCAT<br>GGGTTACAAGGAGAATGTCGATCTGTGGTCTGTGGGATGTATTATGGGTGAAATGGTGTG<br>TCACAAAATCCTCTTTCCCGGCAGGGACTATATTGATCAGTGGAATAAGGTGATCGAGCA<br>GCTGGGAACACCATGCCCAGAGTTTATGAAAAAACTTCAGCCAACCGTGCGAACATATGT<br>GGAAAATCGCCCAAAATATGCAGGCTACAGCTTCGAAAAGCTGTTTCCCGATGTCCTGTT<br>CCCTGCCGATTCTGAGCACAACAAACTGAAGGCTAGCCAAGCTAGGGACCTTCTTAGCAA<br>GATGCTTGTAATCGACGCCTCCAAGAGAATTTCTGTCGACGAGGCTCTGCAACACCCCTA<br>CATTAACGTGTGGTATGATCCTAGCGAAGCAGAGGCGCCTCCTCCCAAAATACCAGATAA<br>GCAGCTGGACGAGCGAGAACACACCATCGAGGAATGGAAGGAGCTTATTTACAAGGAGGT<br>GATGGACCTTGAAGAGCGAACTAAGAACGGCGTGATACGGGGTCAGCCCTCACCGCTGGC<br>TCAGGTTCAACAACACCACCACCATCACCACTGATGA
**GenSmart**	ATGTCCAGAAGCAAGCGAGACAACAACTTCTACAGCGTGGAGATCGGCGACTCAACATTT<br>ACCGTGCTGAAACGGTACCAAAATCTGAAACCTATCGGATCTGGCGCCCAGGGAATCGTG<br>TGCGCCGCTTATGACGCCATCCTCGAGAGGAACGTGGCCATTAAGAAGCTGAGCAGACCC<br>TTCCAAAACCAGACCCACGCCAAGCGGGCCTACAGAGAGCTGGTCCTGATGAAATGCGTG<br>AACCACAAAAACATCATCGGCCTGCTGAACGTGTTCACCCCTCAGAAGAGCCTGGAAGAA<br>TTCCAGGACGTGTACATCGTGATGGAACTTATGGATGCTAATCTGTGCCAGGTCATCCAG<br>ATGGAACTGGACCACGAGAGAATGAGCTACCTGCTGTACCAGATGCTGTGCGGCATCAAG<br>CACCTGCACAGCGCCGGCATCATCCACAGAGATCTGAAGCCCAGCAACATCGTCGTGAAG<br>TCTGATTGCACCCTGAAAATCCTGGACTTCGGCCTGGCTAGAACAGCCGGAACAAGCTTC<br>ATGATGACCCCTTACGTGGTGACAAGATACTACCGGGCCCCTGAGGTGATCCTGGGCATG<br>GGCTATAAGGAAAACGTGGACCTGTGGTCCGTGGGCTGCATCATGGGCGAGATGGTGTGT<br>CACAAGATCCTGTTCCCTGGAAGAGACTACATCGACCAGTGGAACAAGGTGATCGAGCAG<br>CTCGGCACCCCATGTCCTGAGTTTATGAAAAAGCTGCAGCCCACCGTGCGGACCTACGTG<br>GAAAATAGACCTAAGTACGCCGGCTACTCCTTCGAGAAGCTGTTTCCTGATGTTCTGTTC<br>CCCGCCGACAGCGAGCACAACAAACTGAAGGCCTCTCAGGCCAGAGATCTGCTGAGCAAG<br>ATGCTGGTGATCGACGCCAGCAAGCGGATCAGCGTGGACGAGGCCCTGCAACACCCCTAC<br>ATCAACGTGTGGTACGACCCATCTGAGGCCGAGGCTCCTCCTCCAAAGATCCCCGATAAG<br>CAGCTGGATGAGCGGGAACATACCATCGAGGAATGGAAGGAGCTGATCTACAAGGAAGTT<br>ATGGACCTGGAAGAGAGAACAAAGAACGGCGTGATTAGAGGCCAGCCTAGCCCTCTGGCC<br>CAGGTGCAGCAGCACCACCACCATCACCACTGATGA
**COformer**	ATGAGCAGAAGCAAGAGAGACAACAACTTCTACAGCGTGGAGATCGGCGACAGCACCTTC<br>ACCGTGCTGAAGAGATACCAGAACCTGAAGCCTATCGGCAGCGGCGCCCAGGGCATCGTG<br>TGCGCCGCCTACGACGCCATCCTGGAGAGAAACGTGGCCATCAAGAAGCTGAGCAGACCT<br>TTCCAGAACCAGACCCACGCCAAGAGAGCCTACAGAGAGCTGGTGCTGATGAAGTGCGTG<br>AACCACAAGAACATCATCGGCCTGCTGAACGTGTTCACCCCTCAGAAGAGCCTGGAGGAG<br>TTCCAGGACGTGTACATCGTGATGGAGCTGATGGACGCCAACCTGTGCCAGGTGATCCAG

	ATGGAGCTGGACCACGAGAGAATGAGCTACCTGCTGTACCAGATGCTGTGCGGCATCAAG<br>CACCTGCACAGCGCCGGCATCATCCACAGAGACCTGAAGCCTAGCAACATCGTGGTGAAG<br>AGCGACTGCACCCTGAAGATCCTGGACTTCGGCCTGGCCAGAACCGCCGGCACCAGCTTC<br>ATGATGACCCCTTACGTGGTGACCAGATACTACAGAGCCCCTGAGGTGATCCTGGGCATG<br>GGCTACAAGGAGAACGTGGACCTGTGGAGCGTGGGCTGCATCATGGGCGAGATGGTGTGC<br>CACAAGATCCTGTTCCCTGGCAGAGACTACATCGACCAGTGGAACAAGGTGATCGAGCAG<br>CTGGGCACCCCTTGCCCTGAGTTCATGAAGAAGCTGCAGCCTACCGTGAGAACCTACGTG<br>GAGAACAGACCTAAGTACGCCGGCTACAGCTTCGAGAAGCTGTTCCCTGACGTGCTGTTC<br>CCTGCCGACAGCGAGCACAACAAGCTGAAGGCCAGCCAGGCCAGAGACCTGCTGAGCAAG<br>ATGCTGGTGATCGACGCCAGCAAGAGAATCAGCGTGGACGAGGCCCTGCAGCACCCCTTA<br>CATCAACGTGTGGTACGACCCTAGCGAGGCCGAGGCCCCTCCTCCTAAGATCCCTGACAA<br>GCAGCTGGACGAGAGAGAGCACACCATCGAGGAGTGGAAGGAGCTGATCTACAAGGAGGT<br>GATGGACCTGGAGGAGAGAACCAAGAACGGCGTGATCAGAGGCCAGCCTAGCCCTCTGGC<br>CCAGGTGCAGCAGCACCACCACCATCACCACTGATGA

Table S3: Sequences of methods for CREB1

Method	Sequence
**Wild-type**	ATGACCATGGAATCTGGAGCCGAGAACCAGCAGAGTGGAGATGCAGCTGTAACAGAAGCT<br>GAAAACCAACAAATGACAGTTCAAGCCCAGCCACAGATTGCCACATTAGCCCAGGTATCT<br>ATGCCAGCAGCTCATGCAACATCATCTGCTCCCACCGTAACTCTAGTACAGCTGCCCAAT<br>GGGCAGACAGTTCAAGTCCATGGAGTCATTCAGGCGGCCCAGCCATCAGTTATTCAGTCT<br>CCACAAGTCCAAACAGTTCAGATTTCAACTATTGCAGAAAGTGAAGATTCACAGGAGTCA<br>GTGGATAGTGTAACTGATTCCCAAAAGCGAAGGGAAATTCTTTCAAGGAGGCCTTCCTAC<br>AGGAAAATTTTGAATGACTTATCTTCTGATGCACCAGGAGTGCCAAGGATTGAAGAAGAG<br>AAGTCTGAAGAGGAGACTTCAGCACCTGCCATCACCACTGTAACGGTGCCAACTCCAATT<br>TACCAAACTAGCAGTGGACAGTATATTGCCATTACCCAGGGAGGAGCAATACAGCTGGCT<br>AACAATGGTACCGATGGGGTACAGGGCCTGCAAACATTAACCATGACCAATGCAGCAGCC<br>ACTCAGCCGGGTACTACCATTCTACAGTATGCACAGACCACTGATGGACAGCAGATCTTA<br>GTGCCCAGCAACCAAGTTGTTGTTCAAGCTGCCTCTGGAGACGTACAAACATACCAGATT<br>CGCACAGCACCCACTAGCACTATTGCCCCTGGAGTTGTTATGGCATCCTCCCCAGCACTT<br>CCTACACAGCCTGCTGAAGAAGCAGCACGAAAGAGAGAGGTCCGTCTAATGAAGAACAGG<br>GAAGCAGCTCGAGAGTGTCGTAGAAAGAAGAAAGAATATGTGAAATGTTTAGAAAACAGA<br>GTGGCAGTGCTTGAAAATCAAAACAAGACATTGATTGAGGAGCTAAAAGCACTTAAGGAC<br>CTTTACTGCCACAAATCAGATCACCATCACCATCATCACTGATAA
**ExpOptimizer**	ATGACAATGGAAAGCGGAGCCGAAAACCAACAATCCGGAGATGCCGCCGTTACTGAAGCG<br>GAGAACCAGCAGATGACAGTCCAAGCCCAGCCTCAGATTGCTACCCTGGCTCAGGTTTCC<br>ATGCCGGCAGCCCACGCTACGAGTTCAGCACCTACAGTAACACTCGTGCAGTTGCCGAAT<br>GGACAGACTGTACAGGTGCACGGCGTGATCCAGGCCGCTCAGCCAAGTGTTATCCAGTCC<br>CCTCAGGTACAGACCGTGCAGATCAGTACAATCGCAGAGTCCGAAGACTCACAGGAGTCT<br>GTGGACAGTGTAACCGACTCCCAGAAGAGGCGAGAGATACTGTCACGAAGGCCTTCTTAC<br>CGCAAGATCCTGAATGATCTGTCATCCGATGCCCCAGGAGTGCCACGAATCGAGGAGGAG<br>AAGAGCGAAGAGGAAACCTCCGCACCAGCCATTACCACAGTCACAGTCCCCACCCCAATT<br>TATCAGACTTCCTCAGGCCAGTATATCGCCATTACGCAGGGAGGTGCAATCCAGCTTGCA<br>AATAACGGGACCGATGGCGTACAAGGGCTGCAAACACTTACAATGACCAACGCAGCCGCG<br>ACACAGCCAGGAACAACCATACTGCAGTACGCCCAGACCACAGACGGACAGCAGATCCTT<br>GTGCCCAGCAACCAGGTGGTGGTCCAAGCCGCCTCTGGAGACGTGCAGACTTATCAGATT<br>CGCACTGCACCTACTTCCACCATCGCCCCAGGGGTCGTGATGGCTTCCAGCCCTGCTCTG<br>CCAACCCAGCCAGCTGAAGAGGCTGCACGAAAGCGCGAGGTACGCCTGATGAAGAATCGG<br>GAAGCAGCCCGGGAGTGCAGACGAAAGAAAAAGGAGTACGTGAAATGCCTGGAGAATAGG<br>GTGGCTGTGTTGGAGAACCAGAACAAAACCCTGATTGAGGAGTTGAAGGCTCTGAAAGAC<br>CTGTACTGTCACAAGTCAGATCACCACCACCATCACCACTGATGA
**GenSmart**	ATGACAATGGAATCTGGCGCCGAGAATCAACAAAGCGGCGATGCCGCAGTGACAGAGGCT<br>GAGAACCAGCAGATGACCGTCCAGGCCCAGCCCCAGATCGCCACACTGGCCCAAGTGTCC<br>ATGCCTGCCGCCCACGCCACCTCCTCCGCCCCTACCGTGACCCTGGTGCAACTGCCTAAC<br>GGCCAGACCGTGCAGGTGCACGGCGTGATCCAGGCTGCTCAGCCTTCTGTGATCCAGAGC<br>CCTCAGGTGCAGACTGTTCAAATCAGCACCATCGCCGAAAGCGAAGATTCTCAGGAGAGC<br>GTGGACAGCGTGACCGACAGCCAGAAGCGGCGGGAAATCCTGAGCAGACGGCCAAGCTAC<br>AGAAAGATCCTGAACGACCTGAGCAGCGACGCCCCTGGCGTGCCCAGAATCGAGGAGGAG<br>AAGAGCGAGGAAGAGACATCTGCCCCTGCCATCACCACCGTTACCGTGCCTACACCTATC<br>TACCAGACCAGCAGCGGCCAATATATCGCCATTACCCAGGGAGGAGCCATCCAGCTGGCC<br>AACAACGGCACCGATGGCGTGCAGGGCCTTCAGACCCTGACCATGACCAATGCCGCCGCT

	ACACAGCCAGGCACAACAATCCTGCAGTACGCCCAGACCACAGACGGCCAGCAGATCCTC<br>GTGCCCAGCAACCAGGTCGTGGTGCAGGCCGCCAGCGGCGACGTGCAGACCTACCAGATT<br>CGGACCGCCCCTACATCTACAATCGCTCCTGGAGTCGTGATGGCCTCTAGCCCCGCTCTG<br>CCCACCCAGCCTGCTGAAGAGGCCGCCAGAAAAAGAGAGGTGCGGCTGATGAAAAACAGA<br>GAGGCCGCTAGAGAATGTAGAAGGAAGAAGAAAGAGTACGTGAAGTGCCTGGAAAATAGA<br>GTGGCCGTGCTGGAAAACCAGAACAAGACCCTGATCGAGGAGCTGAAGGCCCTGAAGGAC<br>CTGTACTGCCACAAGTCCGATCACCACCACCATCACCACTGATGA
**COformer**	ATGACCATGGAGAGCGGCGCCGAGAACCAGCAGAGCGGCGACGCCGCCGTGACCGAGGCC<br>GAGAACCAGCAGATGACCGTGCAGGCCCAGCCTCAGATCGCCACCCTGGCCCAGGTGAGC<br>ATGCCTGCCGCCCACGCCACCAGCAGCGCCCCTACCGTGACCCTGGTGCAGCTGCCTAAC<br>GGCCAGACCGTGCAGGTGCACGGCGTGATCCAGGCCGCCCAGCCTAGCGTGATCCAGAGC<br>CCTCAGGTGCAGACCGTGCAGATCAGCACCATCGCCGAGAGCGAGGACAGCCAGGAGAGC<br>GTGGACAGCGTGACCGACAGCCAGAAGAGAAGAGAGATCCTGAGCAGAAGACCTAGCTAC<br>AGAAAGATCCTGAACGACCTGAGCAGCGACGCCCCTGGCGTGCCTAGAATCGAGGAGGAG<br>AAGAGCGAGGAGGAGACCAGCGCCCCTGCCATCACCACCGTGACCGTGCCTACCCCTATC<br>TACCAGACCAGCAGCGGCCAGTACATCGCCATCACCCAGGGCGGCGCCATCCAGCTGGCC<br>AACAACGGCACCGACGGCGTGCAGGGCCTGCAGACCCTGACCATGACCAACGCCGCCGCC<br>ACCCAGCCTGGCACCACCATCCTGCAGTACGCCCAGACCACCGACGGCCAGCAGATCCTG<br>GTGCCTAGCAACCAGGTGGTGGTGCAGGCCGCCAGCGGCGACGTGCAGACCTACCAGATC<br>AGAACCGCCCCTACCAGCACCATCGCCCCTGGCGTGGTGATGGCCAGCAGCCCTGCCCTG<br>CCTACCCAGCCTGCCGAGGAGGCCGCCAGAAAGAGAGAGGTGAGACTGATGAAGAACAGA<br>GAGGCCGCCAGAGAGTGCAGAAGAAAGAAGAAGGAGTACGTGAAGTGCCTGGAGAACAGA<br>GTGGCCGTGCTGGAGAACCAGAACAAGACCCTGATCGAGGAGCTGAAGGCCCTGAAGGAC<br>CTGTACTGCCACAAGAGCGACCACCACCACCATCACCACTGATGA

Table S4: Primer sequences for gene EMG1

Method	Primer	Sequence
**Wild-type**	**Vector Forward**	AGCGGCCGCGACTCTAGATC
	**Vector Reverse**	CATGGCGGCAGATCTCCTCGG
	**Insert Forward**	CGAGGAGATCTGCCGCCATGGCCGCGCCCAGTGATG
	**Insert Reverse**	GATCTAGAGTCGCGGCCGCTTATCAGTGATGATGGTGATGGTG
**ExpOptimizer**	**Vector Forward**	AGCGGCCGCGACTCTAGATC
	**Vector Reverse**	CATGGCGGCAGATCTCCTCGG
	**Insert Forward**	CGAGGAGATCTGCCGCCATGGCCGCGCCCAGTGATG
	**Insert Reverse**	GATCTAGAGTCGCGGCCGCTTATCAGTGATGATGGTGATGGTG
**GenSmart**	**Vector Forward**	AGCGGCCGCGACTCTAGATC
	**Vector Reverse**	CATGGCGGCAGATCTCCTCGG
	**Insert Forward**	CGAGGAGATCTGCCGCCATGGCCGCGCCCAGTGATG
	**Insert Reverse**	GATCTAGAGTCGCGGCCGCTTATCAGTGATGATGGTGATGGTG
**COformer**	**Vector Forward**	AGCGGCCGCGACTCTAGATC
	**Vector Reverse**	CATGGCGGCAGATCTCCTCGG
	**Insert Forward**	CGAGGAGATCTGCCGCCATGGCCGCGCCCAGTGATG
	**Insert Reverse**	GATCTAGAGTCGCGGCCGCTTATCAGTGATGATGGTGATGGTG

Table S5: Primer sequences for gene JNK1

Method	Primer	Sequence
**Wild-type**	**Vector Forward**	AGCGGCCGCGACTCTAGATC
	**Vector Reverse**	CATGGCGGCAGATCTCCTCGG
	**Insert Forward**	CGAGGAGATCTGCCGCCATGGCCGCGCCCAGTGATG
	**Insert Reverse**	GATCTAGAGTCGCGGCCGCTTATCAGTGATGATGGTGATGGTG

**ExpOptimizer**	**Vector Forward**	AGCGGCCGCGACTCTAGATC
	**Vector Reverse**	CATGGCGGCAGATCTCCTCGG
	**Insert Forward**	CGAGGAGATCTGCCGCCATGAGCAGAAGCAAGCGTGAC
	**Insert Reverse**	GATCTAGAGTCGCGGCCGCTTATCAGTGATGATGGTGATGGTG
**GenSmart**	**Vector Forward**	AGCGGCCGCGACTCTAGATC
	**Vector Reverse**	CATGGCGGCAGATCTCCTCGG
	**Insert Forward**	CGAGGAGATCTGCCGCCATGAGCAGAAGTAAAAGGGATAACAATTTC-TATAGC
	**Insert Reverse**	GATCTAGAGTCGCGGCCGCTCATCAGTGGTGATGGTGGTGG
**COformer**	**Vector Forward**	AGCGGCCGCGACTCTAGATC
	**Vector Reverse**	CATGGCGGCAGATCTCCTCGG
	**Insert Forward**	CGAGGAGATCTGCCGCCATGTCCAGAAGCAAGCGAGACA
	**Insert Reverse**	GATCTAGAGTCGCGGCCGCTCATCAGTGGTGATGGTGGTGG

Table S6: Primer sequences for gene CREB1

Method	Primer	Sequence
**Wild-type**	**Vector Forward**	AGCGGCCGCGACTCTAGATC
	**Vector Reverse**	CATGGCGGCAGATCTCCTCGG
	**Insert Forward**	CGAGGAGATCTGCCGCCATGACCATGGAATCTGGAGCC
	**Insert Reverse**	GATCTAGAGTCGCGGCCGCTTATCAGTGATGATGGTGATGGTG
**ExpOptimizer**	**Vector Forward**	AGCGGCCGCGACTCTAGATC
	**Vector Reverse**	CATGGCGGCAGATCTCCTCGG
	**Insert Forward**	CGAGGAGATCTGCCGCCATGACAATGGAAAGCGGAGCC
	**Insert Reverse**	GATCTAGAGTCGCGGCCGCTCATCAGTGGTGATGGTGGTGG
**GenSmart**	**Vector Forward**	AGCGGCCGCGACTCTAGATC
	**Vector Reverse**	CATGGCGGCAGATCTCCTCGG
	**Insert Forward**	CGAGGAGATCTGCCGCCATGACAATGGAATCTGGCGCC
	**Insert Reverse**	GATCTAGAGTCGCGGCCGCTCATCAGTGGTGATGGTGGTGG
**COformer**	**Vector Forward**	AGCGGCCGCGACTCTAGATC
	**Vector Reverse**	CATGGCGGCAGATCTCCTCGG
	**Insert Forward**	CGAGGAGATCTGCCGCCATGACCATGGAGAGCGGCG
	**Insert Reverse**	GATCTAGAGTCGCGGCCGCTCATCAGTGGTGATGGTGGTGG

Table S7: Sequences of 5′UTR and 3′UTR

Method	Sequence
**5′UTR**	TCAGATCCGCTAGCGCTACCGGACTCAGATCTCGAGCTCAAGCTTCGAATTCGTCGACTG GATCCGGTACCGAGGAGATCTGCCGCC
**3′UTR**	GCGGCCGCGACTCTAGATCATAATCAGCCATACCACATTTGTAGAGGTTTTACTTGCTTT AAAAAACCTCCCACACCTCCCCCTGAACCTGAAACATAAAATGAATGCAATTGTTGTTGT TAACTTGTTTATTGCAGCTTATAATGGTTACAAATAAAGCAATAGCATCACAAATTTCAC AAATAAAGCA
